# Greatwall depletion from Xenopus oocytes reveals a key role of the cyclin B/CDK1-PP2A-B55 balance in the coordination of meiotic events

**DOI:** 10.1038/s41467-026-73011-5

**Published:** 2026-05-13

**Authors:** Sylvain Roque, Célia Ben Choug, Cedric Hassen Khodja, Suzanne Vigneron, Véronique Legros, Guillaume Chevreux, Benjamin Lacroix, Anna Castro, Thierry Lorca

**Affiliations:** 1 Université de Montpellier, Centre de Recherche en Biologie cellulaire de Montpellier (CRBM)-CNRS, Montpellier, France; 2 Université de Montpellier, Centre de Recherche en Biologie cellulaire de Montpellier-Montpellier Ressources Imagery (MRI) Biocampus, Montpellier, France; 3 ProteoSeine Mass Spectrometry Facility, Université Paris Cité, Institut Jacques Monod, Paris, France

**Keywords:** Kinases, Cell division, Cell signalling

## Abstract

Meiotic progression relies on maintaining a precise balance between cyclin B/CDK1 activity and the phosphatase PP2A-B55. The latter is negatively regulated by the Greatwall kinase (Gwl). In maturing Xenopus oocytes, we show that the loss of Gwl and the subsequent hyperactivation of PP2A-B55 prevents phosphorylation of key proteins involved in spindle formation, chromosome condensation and spindle migration as well as the phosphorylation of Wee1/Myt1 and the APC/C complex. As a consequence, in these oocytes bipolar spindles cannot be formed and migrate to the cortex and chromosomes are partially decondensed preventing meiotic I progression. The APC/C remains inactive impairing cyclin B3 degradation and ultimately preventing Erp1 accumulation. Finally, the c-Mos-MAPK-Rsk1/2 pathway fails to activate due to the absence of c-Mos as a consequence of its improper degradation. Overall, our findings reveal a crucial role of Gwl in the coordination and progression of meiotic divisions.

## Abstract (web summary)

This study highlights how Greatwall coordinates the progression of meiosis by regulating the activity of PP2A-B55. Here, the authors show that in the absence of Greatwall, the c-Mos protein is degraded, cyclin B3 remains stable, and Erp1 fails to accumulate.

## Introduction

Meiosis is a specialized cell division process that reduces the chromosome number by half, generating genetically distinct haploid gametes essential for sexual reproduction. Additionally, meiosis introduces genetic diversity through the recombination and segregation of homologous chromosomes^1^. This process plays a critical role in sexual reproduction and is tightly regulated by the coordinated activity of kinases, including Protein Kinase A (PKA) and cyclin B1/B2/CDK1 (hereafter referred to as ‘cyclin B/CDK1’), which orchestrate key protein phosphorylation events and ensure timely cell cycle transitions.

PKA activity is essential at early stages of meiotic division to establish a prophase I arrest, ensuring that oocytes remain fully grown and developmentally competent for fertilization. This arrest is mediated by PKA via two parallel mechanisms: (1) suppressing mRNA translation of key cell cycle regulators, and (2) downregulating cyclin B/CDK1 activity through Myt1-dependent phosphorylation of CDK1 at its inhibitory residues (T14 and Y15). Next, progesterone (Pg) releases prophase I arrest by downregulating PKA, inducing the activation of Cdc25 and cyclin B/CDK1, and promoting meiotic resumption.

Oocytes then progress into anaphase I and exit this phase of meiosis by inducing a partial degradation of cyclin B1/B2. The incomplete loss of these cyclins guarantees the presence of a residual cyclin B/CDK1 activity essential for preventing an intermediate S phase to occur between meiosis I and II and ensuring a correct first reductional division².

Finally, at the end of meiosis, oocytes accumulate Emi2/Erp1 protein, an Anaphase Promoting Complex/Cyclosome (APC/C) inhibitor that stabilizes cyclin B1/B2, keeping cyclin B/CDK1 activity high and maintaining metaphase II until fertilization. Notably, Erp1 expression starts from GVBD^2^, but its accumulation is prevented by cyclin B3/CDK1-dependent phosphorylation that promotes its ubiquitination and degradation. At the end of meiosis I, cyclin B3 is degraded by the APC/C^3,4^, and this negative modulation is lifted, permitting the stabilization of Erp1 by c-Mos-MAPK-Rsk1/2-dependent phosphorylation and thereby enabling APC/C inhibition and metaphase II arrest^5^.

Besides the role of kinases, the regulation of phosphatases, and notably of PP2A-B55, is also crucial to establish a correct timing of protein phosphorylation and meiotic progression^6^. The mechanisms controlling PP2A-B55 activity have been largely studied in the context of mitotic division. In this process, PP2A-B55 is controlled by the Gwl kinase, also known as MASTL (Microtubule-Associated Serine/Threonine Kinase-Like) in humans. Gwl is turned off during interphase when PP2A-B55 remains active and dephosphorylates key mitotic regulators such as Myt1, Wee1, and Cdc25, thereby preventing cyclin B/CDK1 activation, substrate phosphorylation, and entry into mitosis^7–9^. At mitotic entry, Gwl kinase is turned on and phosphorylates its substrates ENSA and Arpp19, converting them into potent inhibitors of PP2A-B55 and enabling the subsequent phosphorylation of Myt1, Wee1, and Cdc25, finally activating cyclin B/CDK1^10,11^. Unlike mitosis, the role of Gwl kinase in meiotic maturation is not fully understood. In this study, we used the TRIM-Away technique to selectively degrade Gwl in Xenopus oocytes and assess its function during meiosis. Our results show that Gwl depletion leads to sustained PP2A-B55 activity, which in turn prevents phosphorylation and activation of proteins involved in spindle formation and chromosome condensation, as well as the phosphorylation and activation of the APC/C complex and the c-Mos-MAPK-Rsk1/2 pathway. As a result, the spindle cannot be formed, and the chromosomes remain undercondensed. Moreover, the APC/C remains inactive, leading to the accumulation of cyclin B3 and the continuous degradation of its inhibitor Erp1. Moreover, c-Mos is not accumulated, preventing downstream activation of Rsk1/2. These combined defects severely impair meiotic progression. Together, our findings reveal that Gwl, through PP2A-B55 regulation, is essential for orchestrating the meiotic program by coordinating the phosphorylation of key proteins involved in spindle formation and chromosome condensation as well as the activation of the APC/C and of the c-Mos pathway.

## Results

### Depletion of the Gwl kinase does not prevent meiotic resumption in Xenopus oocytes

**Fig. 1:**
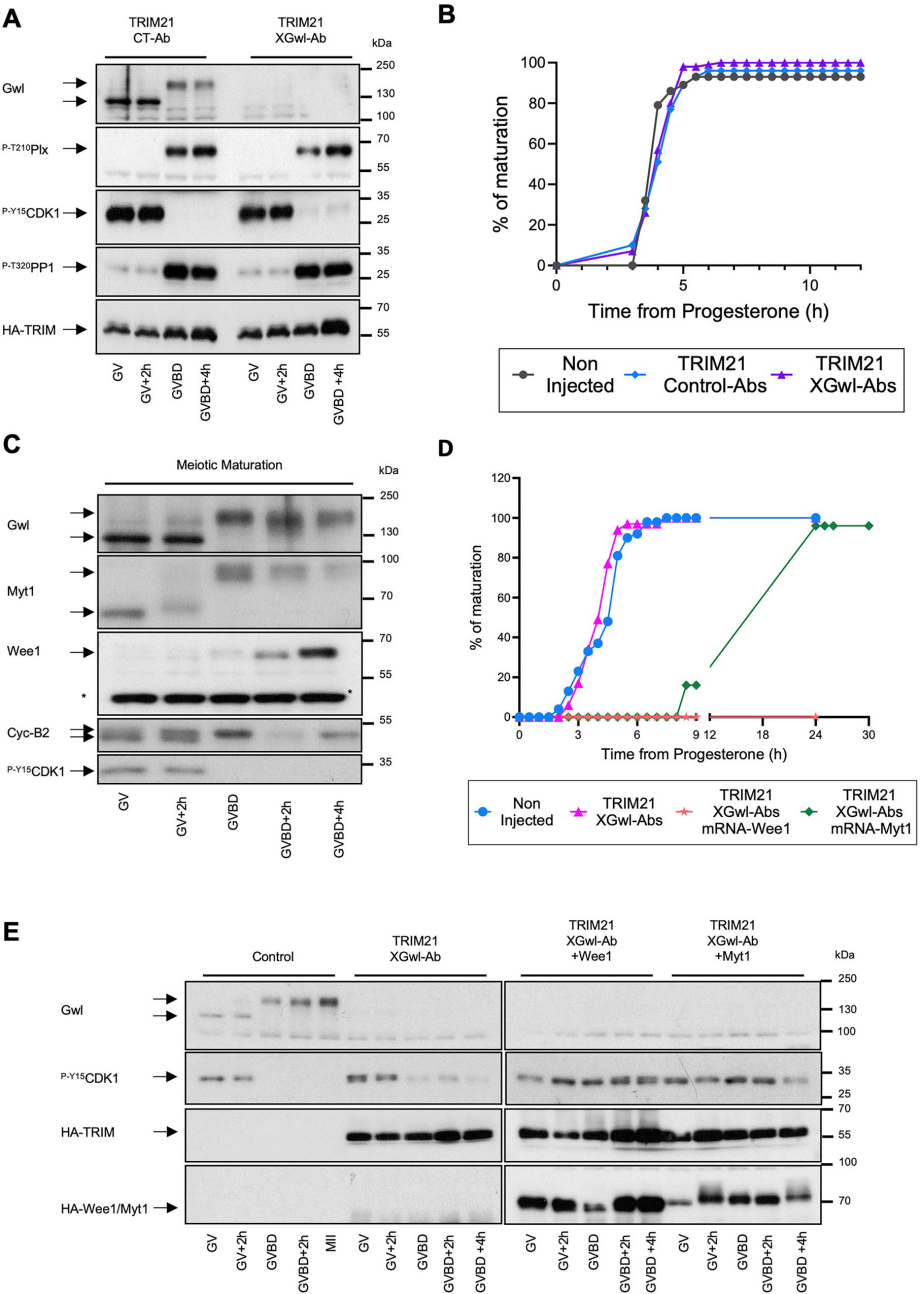
Depletion of the Gwl kinase does not prevent meiotic resumption in Xenopus oocytes. **A** Prophase I arrested oocytes were injected with HA-TRIM21 mRNA and either control or Xenopus anti-Gwl antibodies (XeGwl-Abs). Sixteen hours later, progesterone was added and oocytes were recovered at prophase I (GV), 2 h upon Pg addition (GV + 2 h), at GVBD and 4 h upon GVBD (GVBD + 4 h) and analysed by western blot to detect Gwl and HA-TRIM21 proteins, as well as the phosphorylation status of Plx1 (T210), the phosphatase PP1 (T320), and Cdk1 (P-Y15). **B** Germinal Vesicle Breakdown (GVBD, marked by the appearance of a white spot in the animal pole of the oocytes) was monitored over time in oocytes in (**A**). **C** Oocytes arrested in prophase I were treated with Pg, and the levels of Gwl, Wee1, Myt1, cyclin B2, and the phosphorylation of CDK1 on Y15 were measured at the indicated timepoints. **D** Prophase I-arrested oocytes were either injected or not (blue) with HA-TRIM21 mRNA and XeGwl Abs (magenta), along with Wee1 (red) or Myt1 (Green) mRNAs. Sixteen hours later, oocytes were treated with progesterone, and GVBD was monitored over time. **E** Oocytes in (**D**) were removed at the indicated time-points and analysed by Western blot to examine the levels of HA-TRIM21, HA-Wee1, HA-Myt1, Gwl, and phosphorylation of Cdk1 on Y15. Data (graphs and Western blots) are representative of at least three different experiments.

In order to assess the role of Gwl in meiotic progression, we first checked the expression pattern of this kinase during meiotic maturation in Xenopus oocytes. As shown in Fig. 1A, Gwl protein is stably expressed in oocytes at all stages, including fully-grown stage VI immature oocytes (Prophase I-arrested oocytes or Germinal Vesicle: GV), maturing oocytes 2 h upon Pg stimulation (GV + 2 h), oocytes upon Germinal Vesicle Breakdown (GVBD), and metaphase II-arrested oocytes (MII). As expected, this kinase displays a delayed electrophoretic mobility at GVBD and MII oocytes, indicative of its phosphorylation and activation. Complete Gwl phosphorylation is concomitant with Plx1 and cyclin B/CDK1 activation, as indicated by Plx1 phosphorylation on T210 activatory site and CDK1 dephosphorylation of Y15 inhibitory residue. We next depleted Gwl from prophase I-arrested oocytes using the TRIM-Away approach^12^. This technique employs the TRIM21 protein, an E3 ubiquitin ligase that associates to antibody-bound targets and facilitates their degradation via the proteasome^13^. As expected, the microinjection of these oocytes with the mRNA encoding HA-tagged TRIM21 and anti-Xenopus Gwl antibodies promoted the complete depletion of Gwl, an effect that was not observed when control anti-GST instead of Gwl antibodies were used (Fig. 1A). We next stimulated these oocytes with Pg and scored the presence of a white spot in the animal pole as a marker of GVBD. Both control and Gwl-depleted (ΔGwl) oocytes presented a white spot at 4 h upon Pg addition, although this spot was larger in the latter group of oocytes that rapidly degenerated in a few hours (Figs. 1B and S1). To confirm that this white spot was associated with meiotic resumption, we checked the phosphorylation of CDK1 on Y15, of Plx1 on T210, and of the endogenous CDK1 substrate T320 of PP1. No differences in the phosphorylation patterns of CDK1 on Y15, of Plx1 on T210, and of the endogenous CDK1 substrate T320 of PP1 were observed between control (CT) and ΔGwl oocytes, indicating that Gwl depletion enables normal cyclin B/CDK1 activation and meiotic resumption (Fig. 1A).

Intriguingly, unlike Gwl-devoid immature oocytes, it has been shown that Gwl depletion in G2 first embryonic Xenopus egg extracts prevents cyclin B/CDK1 activation by maintaining Y15 phosphorylation of CDK1^9^. A major difference between G2 egg extracts and stage VI oocytes is that Wee1 phosphatase is not expressed in stage VI whereas it is already present in first embryonic division^14^. A putative explanation for this discrepancy could be that, because of the absence of Wee1, immature oocytes would be unable to fully phosphorylate CDK1 on Y15 in the absence of Gwl. To investigate this hypothesis, we overexpressed either Myt1 or Wee1 in stage VI oocytes and checked their effect in meiotic maturation. As previously described, while Myt1 kinase was already present in immature oocytes, we observed the expression of Wee1 in control non-treated oocytes from 2 h after GVBD concomitantly with the partial drop of cyclin B2 typically observed in the oocytes between MI-MII (Fig. 1C). Interestingly, the overexpression of Myt1 in ΔGwl oocytes promoted 100% of maturation **albeit with a drastic delay** and residual Y15 phosphorylation in CDK1 (Fig. 1D, E). Conversely, Wee1 overexpression, completely blocked meiotic resumption, which suggests that the absence of Wee1 in Gwl-devoid oocytes enables meiotic progression despite the presence of a hyperactive PP2A-B55 phosphatase.

### Gwl depletion induces drastic changes in the protein phosphorylation pattern of meiotic oocytes

**Fig. 2:**
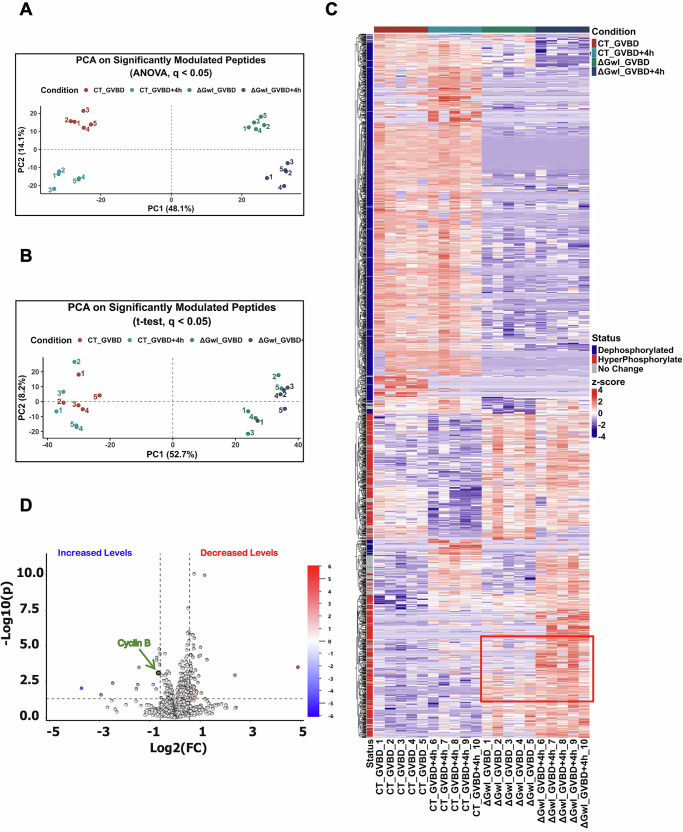
Gwl depletion induces drastic changes in the protein phosphorylation pattern of meiotic oocytes. **A** Principal component analysis (PCA) of significantly modulated phosphopeptides. Plot visualizing the distribution of biological replicates along the first two principal components based on phosphopeptide intensities significantly altered across conditions (ANOVA with FDR correction, *q* < 0.05). Four experimental conditions are represented: control GVBD (red), control GVBD + 4 h (blue), ΔGwl GVBD (green), and ΔGwl GVBD + 4 h (grey). PC1 (48.2%) primarily separates control from ΔGwl samples, while PC2 (14.1%) distinguishes between developmental stages. Each point represents an individual biological replicate. Dashed lines indicate PC1 = 0 and PC2 = 0 axes. **B** PCA of significantly modulated phosphopeptides (*t*-test) of GVBD and GVBD + 4 h stages combined within each treatment group (control vs ΔGwl). Phosphopeptides significantly modulated (*q* < 0.05, two-group *t*-test with FDR correction) were used for log₂-transformed phosphorylation intensities. Each point represents a biological replicate, with control samples depicted in red (CT GVBD) and blue (CT GVBD + 4H) and ΔGwl samples in green (ΔGwl GVBD) and grey (ΔGwl GVBD + 4H). The percentage of variance explained by PC1 and PC2 is shown in parentheses. Dashed lines indicate PC1 = 0 and PC2 = 0. **C** Heat map illustrating the dynamic changes in differentially phosphorylated peptides between control (GVBD and 4 h post GVBD) and Gwl-devoid oocytes (GVBD and 4 h post GVBD). **D** Volcano plot representing protein abundances between oocytes injected with control and XeGwl antibodies. Log2(Fold Change (FC) of control vs Gwl antibody injection) in *x*-axis; -Log10(*p*-value) on *y*-axis. Vertical lines: [logFC] = 1; horizontal line: *q* = 0.05. Indicated are the levels of cyclin B. Statistical tests on MS data were two-sided, *p*-values were used for data interpretation, but adjusted *p*-values according to the Benjamini-Hochberg procedure (*q*-values) were also calculated and provided for information in code availability.

To further characterize the role of Gwl in meiotic progression, we performed a label-free phosphoproteomic analyses in oocytes at GVBD and 4 h later (GVBD + 4 h), and we examined the phosphorylation changes induced by Gwl loss and the subsequent reactivation of PP2A-B55. We analysed four groups, GVBD and GVBD + 4 h depleted (ΔGwl) or not (CT) of Gwl. For each group, we used five replicates of 10 control and 10 ΔGwl oocytes coming from different frog females (Fig. S2), and we conducted a LC-MS/MS-based phosphoproteomic analysis. Data were first analysed using a Principal Component Analyses (PCA) for the four different groups to assess factors driving phosphorylation variation. PCA outputs revealed that principal component 1 (PC1) produced two clearly separated groups corresponding to control and Gwl depleted oocytes (Fig. 2A), indicating that Gwl depletion induced major phosphorylation differences, while principal component 2 (PC2) separated GVBD from GVBD + 4 h oocytes, suggesting that developmental stage has a minimal effect on the phosphoproteome. In a second PCA, the temporal variable was removed by combining the two stages of control and ΔGwl replicates. Again, PC1 clearly separated control from ΔGwl oocytes, highlighting the strong effect of Gwl depletion in protein phosphorylation, while PC2 only captured residual variability (Fig. 2B).

We next checked the global proteome and phosphoproteome changes induced by Gwl depletion in meiotic oocytes. As depicted in the heatmap analysis of Fig. 2C, the loss of Gwl promoted both dephosphorylation (upper heatmap part) and hyperphosphorylation (lower heatmap part) at a similar proportion (49.6 % vs 51.7, respectively) in numerous peptides of both GVBD and GVBD + 4 h groups. Although some differences were observed between these two stages (Fig. 2C, red square), dephosphorylation/hyperphosphorylation was consistently widespread across all five biological replicates. Interestingly, we did not observe major changes in protein levels, with only 46 from a total of 4371 identified proteins displaying differential expression between control and ΔGwl oocytes (Fig. 2D). Interestingly, this short list included cyclin B, whose abundance was significantly higher in ΔGwl oocytes. These data suggest that phosphorylation modifications were not a consequence of changes in protein abundance and support a robust and reproducible effect of Gwl depletion in phosphoprotein pattern modification.

**Fig. 3:**
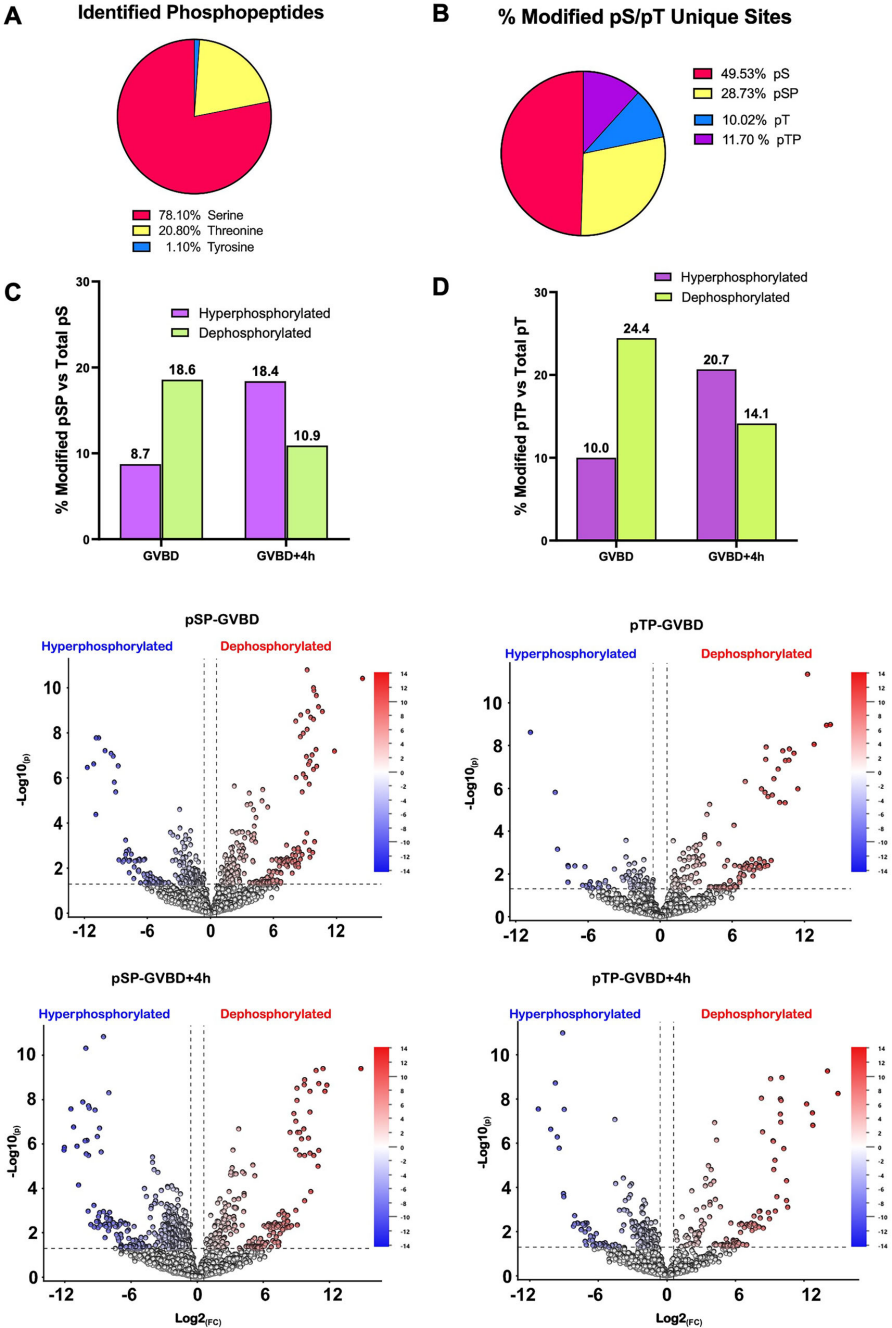
Analysis of differentially phosphorylated peptides in Gwl depleted oocytes. **A** Representation of the percentage of serine (red), threonine (yellow), and tyrosine (blue) phosphosites identified in the label-free phosphoproteome. **B** Representation of the percentage of non-SP/TP (red/blue) and SP/TP (yellow/violet) unique sites differentially dephosphorylated (*q* < 0.05) in control vs Gwl depleted oocytes of both GVBD and GVBD + 4 h conditions combined. **C** The percentage of unique SP consensus sites at GVBD and GVBD + 4 h phosphorylated (violet) or dephosphorylated (green) upon Gwl depletion respect to total pS sites was represented. Volcano plots representing phosphorylation changes in pSP sites between control and Gwl antibody injected oocytes at GVBD and GVBD4h. Log2(Fold Change (FC) of control vs Gwl antibody injected oocytes) in *x*-axis; -Log10(p-value) on *y*-axis. Vertical lines: [logFC] = 1; horizontal line: *q* < 0.05. Statistical tests on MS data were two-sided, *p*-values were used for data interpretation, but adjusted *p*-values according to the Benjamini-Hochberg procedure (*q*-values) were also calculated and provided for information in code availability. **D** As for (**C**) except that pTP instead of pSP was analysed.

Our phosphoproteomic data identified 15.386 phosphopeptides from 5.300 proteins, from which 78.1% contained phospho-serine (pS), 20.8% phospho-threonine (pT), and 1.1% phospho-tyrosine (pY) (Fig. 3A). From these phosphopeptides, we identified 12987 pS/pT unique sites with 20% displaying either increased or decreased phosphorylations upon Gwl depletion. Interestingly, we observed that 40.4% of the differential phosphorylations were performed in cyclin/CDK S/T-P consensus motifs indicating that 59.5% of pS/pT modifications were likely performed by other kinases/phosphatases indirectly modified by the established cyclin B/CDK1/PP2A-B55 equilibrium (Fig. 3B). Our data additionally show a similar proportion of pTP and non-pTP differentially phosphorylated sites whereas pSP represented half of the non-pSP motif suggesting that phosphorylation changes were preferentially performed in the TP motifs.

Interestingly, at GVBD, ΔGwl oocytes displayed a higher proportion of dephosphorylated relative to hyperphosphorylated pSP and pTP sites (Fig. 3C, D). This difference was more pronounced for pTP sites, consistent with the known preference of PP2A-B55 for these motifs^15,16^. Phosphorylation/dephosphorylation distribution was inversed in SP/TP cyclin B/CDK1 consensus motifs at GVBD + 4 h with a drastic increase in phosphorylation, notably in pSP consensus sites. Thus, Gwl depletion results in a marked dephosphorylation of pTP motifs at GVBD and hyperphosphorylation of pSP at GVBD + 4 h. Although alternative explanations cannot be ruled out, the observed dephosphorylation at GVBD could be attributable to a delay or failure of cyclin B/CDK1 to phosphorylate its substrates in the presence of hyperactive PP2A-B55 during early meiotic stages. Conversely, hyperphosphorylation at GVBD + 4 h may stem from an hyperactivation of cyclin B/CDK1 due to the accumulation of cyclin B and/or from the sequential activation of additional kinase cascades typically suppressed under control conditions.

To further investigate the kinases/phosphatases involved in pSP/TP and non-pSP/TP modifications, we performed IceLogo analysis^17^ (Fig. S3). The first observation was an enrichment of T respect to S among both differentially dephosphorylated and hyperphosphorylated sites. Unsurprisingly, enriched amino acid sequences in dephosphorylated pSP/TP residues agrees PP2A-B55 consensus motifs, whereas dephosphorylated non-pSP sites were enriched in RxxS sequence, a motif targeted by Phactr1/PP1 phosphatase^18^. Finally, non-pTP dephosphorylated residues displayed an enrichment of a T containing multi-site phospho-motif (STSxxS), an enriched sequence also compatible with PP1-induced dephosphorylation^19^. Proteins hyperphosphorylated in pSP/TP residues present the perfect consensus motif of cyclin/CDK (S/TPxK/R), while hyperphosphorylation in non-TP sites revealed an enrichment of threonine sites surrounded by multi-SP motifs, consistent with phosphorylation mediated by cyclin/CDK/Cks complex^20^. Lastly, non-SP hyperphosphorylated sites displayed an enrichment in RRxS, the consensus motif of PKA. Thus, phosphorylation differences induced by Gwl depletion are likely induced by PP2A-B55 and PP1 phosphatases and cyclin/CDK and PKA kinases, four enzymes already known to play a key role in meiotic progression.

Finally, we explored the potential biological functions of the differentially phosphorylated proteins using Kyoto Encyclopedia of Genes and Genomes (KEGG) pathway analysis using String database^21^ (https://string-db.org) (Fig. S4). This analysis revealed that dephosphorylated proteins are mainly involved in (1) mRNA transport pathway (including members of the NUP complexes and the eukaryotic initiation factor (Eif4a/b)), (2) progesterone-mediated oocyte meiosis (with several members of the MAPK pathway and proteins involved in meiotic progression such as Cdc25 and APC1), and (3) proteins regulating actin cytoskeleton (Wasf, Pxn, Ptk, or the RhoGap *Arhgap)*. KEGG analysis of hyperphosphorylated proteins revealed an enrichment in (1) mTOR pathway (MAPK members), (2) mRNA transport, and (3) DNA replication (including Pol2A, MCM4, Polε, or the PCNA loader subunit Rfc1). Interestingly, components of the mRNA transport pathway showed enrichment in both hyperphosphorylated and dephosphorylated residues, albeit on distinct sites (Nup35, Tpr, Ranbp2, Eif4b, and Tacc3). Unsurprisingly, all these protein families play an essential role in meiotic maturation by controlling protein translation, spindle formation, and cell cycle progression.

### Gwl depletion prevents c-Mos and Erp1 accumulation in maturing oocytes

**Fig. 4:**
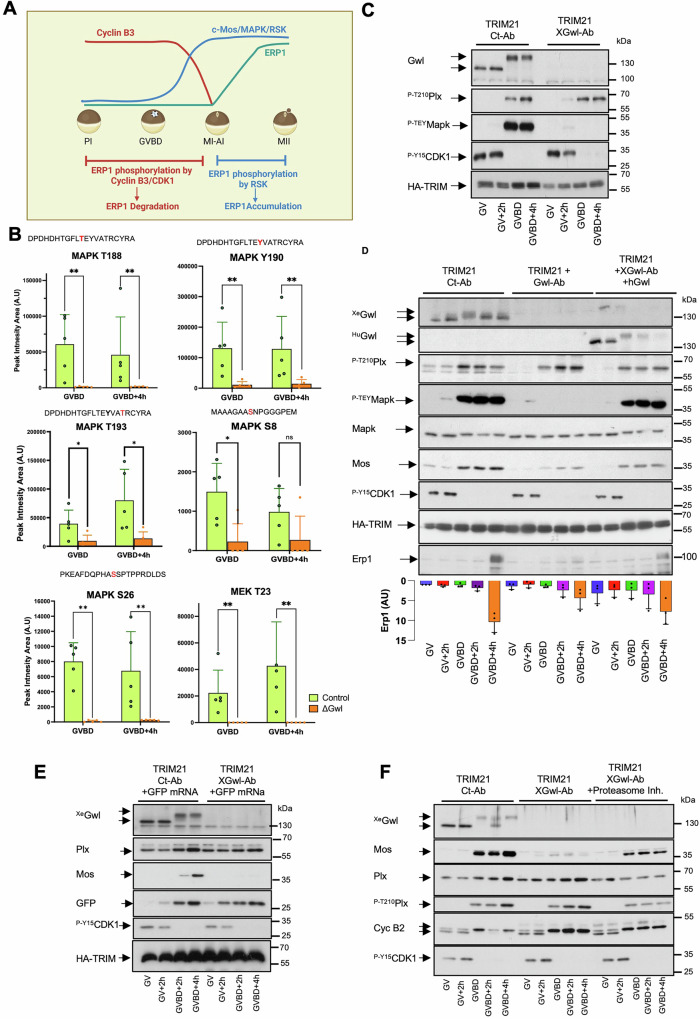
Gwl depletion prevents c-Mos and Erp1 accumulation in maturing oocytes. **A** Scheme illustrating the mechanisms controlling Erp1 stability during meiotic maturation. Created with BioRender.com. Created in BioRender. https://BioRender.com/3jpud55. **B** Peak intensity area of the indicated phosphosites on MAPK and in MEK control (Green) and ΔGwl (Orange) oocytes obtained by mass spectrometry in GVBD and GVBD+4h conditions. The position of the indicated phospho-sites in the TEY motif is indicated in red. Data are expressed as mean ± Standard Deviation (SD) from five independent replicates. Statistical analyses using two-sided non-parametric Mann–Whitney *U* test were performed. *p < 0.05; **p < 0.008; ****p < 0.0001. *n* = 5. **C** Prophase I arrested oocytes were injected with HA-TRIM21 mRNA and control or Gwl antibodies. Sixteen hours later, oocytes were treated with Pg to induce meiotic maturation, and the levels and phosphorylation of the indicated proteins analysed by western blot at the specified time points. Data are representative of at least three different experiments. **D** Prophase I oocytes were injected with HA-TRIM21 mRNA and control or Gwl antibodies. Sixteen hours later half of the oocytes devoid of Gwl were injected with the human Gwl protein (hGwl). One hour later oocytes were treated with Pg and immunoblotted at indicated time points to detect Xenopus (^Xe^Gwl) and Human Gwl (^Hu^Gwl), MAPK, c-Mos, Erp1, and HA-TRIM21 protein levels and the phosphorylation of Plx1 on T210, MAPK on the TEY motif, and of CDK1 on Y15. Erp1 western blot signal from three different experiments was quantified and represented as mean ± SD. **E** Oocytes were injected with HA-TRIM21 and a GFP mRNA containing the 5’ and 3’ UTR of c-Mos, as well as with Ct or XeGwl Abs, and 16 h later treated to Pg. At the indicated time-points, oocytes were recovered and used for western blot analysis to detect the indicated proteins/phosphoproteins. Data are representative of at least three different experiments. **F** Oocytes were injected with HA-TRIM21 mRNA and Ct or XeGwl Ab, and 16 h later, the proteasome inhibitor epoxomicin was added or not to the media at a final dose of 100 µM. One hour later, Pg was supplemented, and oocytes recovered for western blot analysis at the indicated time-points. Data (Western blots) are representative of three different experiments.

To further investigate the role of Gwl in meiotic progression, we focused our interest in those dephosphorylated proteins known to be involved in the control of this cellular process. One major pathway controlling meiotic maturation is the c-Mos/MEK1/MAPK1 cascade. During meiotic progression, c-Mos is translated at GVBD, promoting the activation of the MAP Kinase Kinase 1, MEK1, and the final phosphorylation of MAPK1^22^. Once activated, MAPK1 turns on Rsk1/2 (hereafter referred to as “Rsk”) which then phosphorylates and stabilizes Erp1, resulting in the subsequent inhibition of the APC/C and the establishment of the metaphase II arrest^23^ (Fig. 4A). In our phosphoproteomic data, we identified T23 of MEK1 and S8, S26, T188, Y190 and T193 of MAPK1 as being dephosphorylated in Gwl-devoid Pg-treated oocytes (Fig. 4B). Interestingly, T188 and Y190 belong to the TEY motif of MAPK1 and its double T188/Y190 phosphorylation promotes its activation^24^. Moreover, phospho-T193 is an autophosphorylation site acting as a feedback autoregulation to restrict MAPK1 activity^25^. We thus used specific phospho-antibodies against the TEY motif to confirm Y190 and T188 dephosphorylation upon Gwl depletion. As expected, phosphorylation of this motif was observed upon Pg addition in control oocytes from GVBD concomitantly with the activation of Plx1 (T210 phosphorylation) and of cyclin B/CDK1 (Y15 dephosphorylation), whereas, TEY phosphorylation was undetectable in Gwl depleted oocytes despite these oocytes activated cyclin B/CDK1. Interestingly, TEY dephosphorylation was associated with the absence of c-Mos and Erp1, indicating that the c-Mos/MEK1/MAPK1 cascade cannot be turned on in the absence of Gwl (Fig. 4D). To confirm the specificity of our findings, we performed a rescue experiment by co-injecting the human Gwl (huGwl) protein together with HA-tagged TRIM21 mRNA and anti-Xenopus Gwl antibodies. Because huGwl only shares 53% amino acid sequence identity with its Xenopus orthologue, it is barely recognized by anti-Xenopus Gwl antibodies and thus, is poorly targeted by TRIM21.

Moreover, the microinjection of huGwl restored MAPK phosphorylation and c-Mos and Erp1 levels, confirming that this phenotype was specifically induced by Gwl depletion (Fig. 4D).

**Fig. 5:**
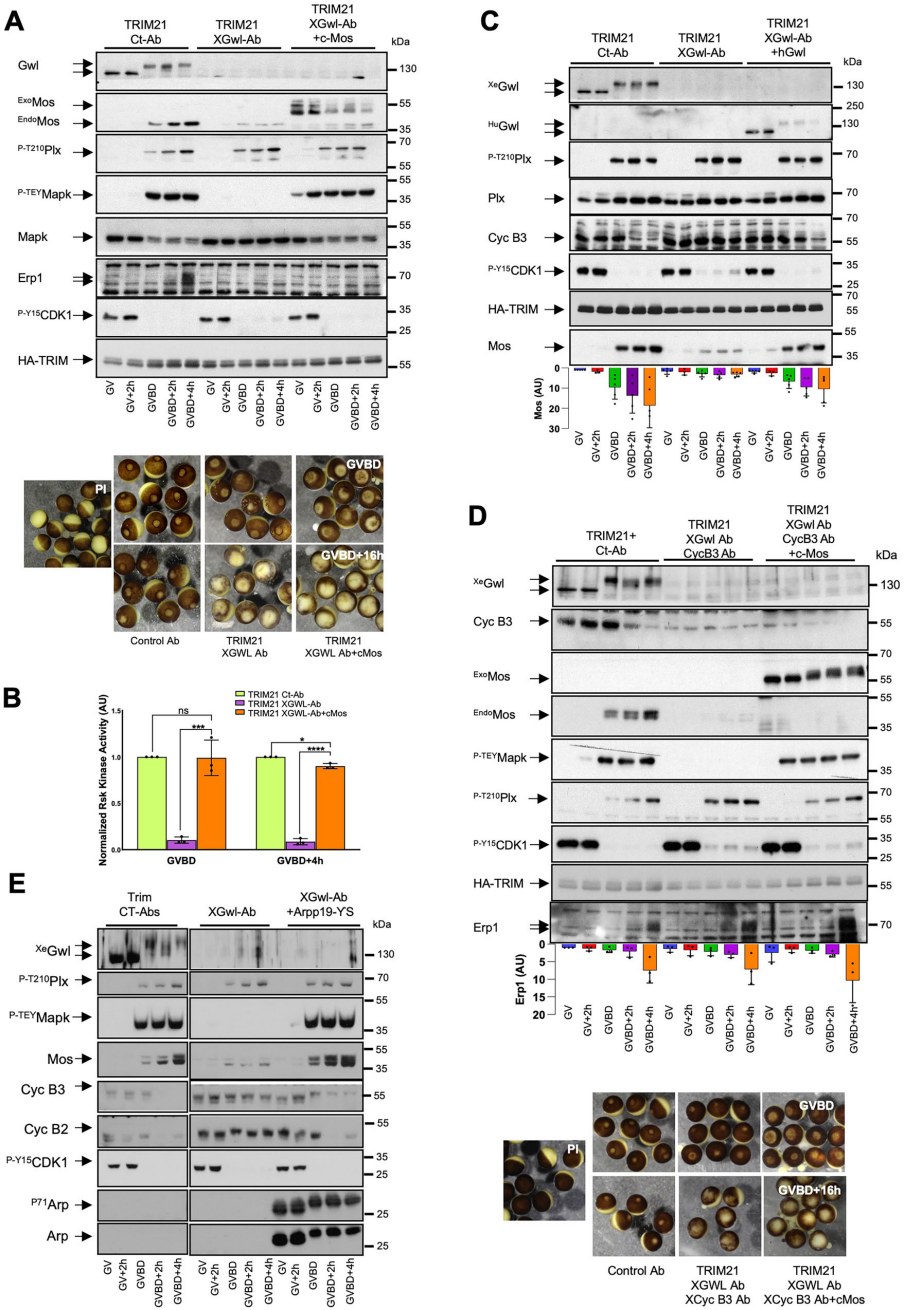
Xenopus oocytes lacking Gwl fail to accumulate c-Mos and Erp1 proteins and to degrade cyclin B3. **A** Prophase I oocytes were injected with HA-TRIM21 mRNA and control or XeGwl Abs. Sixteen hours later, half of Gwl-devoid oocytes were injected with Xenopus c-Mos protein and subsequently treated with Pg. Oocytes were then recovered at the indicated time-points and immunoblotted to detect Gwl, endogenous and overexpressed tagged c-Mos, MAPK, Erp1, and HA-TRIM21 protein levels as well as regulatory phosphorylation of Plx1 MAPK and Cdk1. Pictures of the treated oocytes at the different time-points are presented. Data are representative of at least three different experiments. **B** P90Rsk kinase activity was measured in vitro using the Kemptide peptide as a substrate in control (Green), ΔGwl(violet), and ΔGwl + cMos(orange) oocytes and represented as the percentage of maximal activity. Data are expressed as mean ± Standard Deviation (SD) from three independent replicates Statistical analyses using two-sided non-parametric Mann–Whitney *U* test were performed. *p < 0.01; ***p < 0.0005; ****p < 0.0001. *n* = 3. **C** Prophase I oocytes were injected with HA-TRIM21 mRNA and control or XeGwl antibodies. Sixteen hours later, half of the oocytes lacking the Gwl protein were injected with huGwl protein and subsequently treated with Pg and analysed by Western blot at the indicated time points to determine the phosphorylation and the levels of the specified proteins. c-Mos western blot signal was quantified in three different experiments and represented as a mean ± SD. **D** Xenopus prophase I arrested oocytes were injected with HA-TRIM21 mRNA and control or both XeGwl and cyclin B3 antibodies. 16 h later, half of the oocytes lacking Gwl and Cyclin B3 proteins were injected with Xenopus c-Mos protein. Upon treatment with Pg, a sample of these oocytes was removed at the indicated time points and immunoblotted to detect the levels and the phosphorylation of the specified proteins. Erp1 western blot signal was quantified in three different experiments and represented as a mean ± SD. Pictures of the treated oocytes at the different time points are shown. **E** HA-TRIM21 and Ct or XeGwl Ab injected oocytes were or not microinjected with thio-(S71)-XArpp19 protein, and 1 h later supplemented with Pg. Oocytes were then recovered for Western blot at the indicated time-points. Data are representative of at least three different experiments.

We next sought to investigate the underlying causes of c-Mos loss in Gwl-depleted oocytes. To this, we first checked the effect of Gwl degradation in c-Mos translation using a mRNA reporter coding for GFP and containing the 5’ and 3’ UTR regions of c-Mos. This mRNA was co-injected with HA-TRIM21 mRNA and XGwl or control antibodies into the oocytes, and upon Pg addition, c-Mos and GFP levels were measured during meiotic progression. As expected, c-Mos protein was present in ΔCt, but not in ΔGwl oocytes, however, GFP levels were identical in both types of oocytes, indicating that c-Mos translation was not affected by the depletion of Gwl (Fig. 4E). We thus investigated whether c-Mos loss could be induced by protein ubiquitination and degradation. To address this, we examined the effect of inhibiting the proteosome by the specific inhibitor expoxomicin (Fig. 4F). Interestingly, c-Mos levels were restored in ΔGwl oocytes during meiotic progression, supporting that Gwl depletion induces c-Mos degradation and prevents subsequent MEK1, MAPK1, and Rsk activation during meiotic maturation. Moreover, since Erp1 accumulation depends on c-Mos (Fig. 4A), this degradation could also be responsible of the absence of Erp1. If this is the case, the overexpression of c-Mos in Gwl-depleted oocytes should restore MEK1/MAPK1/Rsk cascade activation and the final phosphorylation and stabilization of Erp1. However, surprisingly, the co-injection of c-Mos protein with HA-TRIM21 mRNA and XGwl antibodies rescued MAPK1 phosphorylation but was unable to restore Erp1 protein levels (Fig. 5A).

Erp1 lack in c-Mos co-injected oocytes could be due to the incapacity of the ectopic c-Mos protein to provide enough MAPK1 activity to turn on Rsk and to trigger Erp1 phosphorylation and accumulation, or to the dysregulation in Gwl depleted oocytes of an additional pathway participating to Erp1 stability. To address these hypotheses, we evaluated Rsk activity under three experimental conditions: control, ΔGwl, and ΔGwl + c-Mos. Our results clearly indicate that exogenous c-Mos effectively activates Rsk, however, this activation is unable to stabilize Erp1 in the absence of Gwl (Fig. 5B). Our results suggest that Gwl might regulate another pathway leading to Erp1 stabilization.

### Depletion of Greatwall from Xenopus oocytes impairs cyclin B3 degradation

Recent studies showed that cyclin B3-CDK1 kinase phosphorylates Erp1 from prophase I to anaphase I, promoting its degradation by the proteasome and preventing the arrest of oocytes during metaphase I^4^. At anaphase I, this phosphorylation-dependent destabilization mechanism of Erp1 is halted by the degradation of cyclin B3. Thus, Erp1 stability is controlled by both Rsk- and cyclin B3-CDK1-dependent phosphorylation (Fig. 4A). Data above demonstrate that Rsk activation is not sufficient to stabilize Erp1. Hence, we next checked whether Gwl depletion could perturb cyclin B3 stability during oocyte maturation. As expected, cyclin B3 is degraded at anaphase I in control oocytes, once MAPK1, cyclin B/CDK1, and Plx-1 are activated. However, this protein was stabilized throughout meiosis in Gwl depleted oocytes (Fig. 5C). This effect is specific since the addition of huGwl restores cyclin B3 degradation. These findings suggest that, besides the incapacity to stabilize c-Mos, the absence of Gwl in the oocytes prevents cyclin B3 proteolysis, a double effect that will prevent Erp1 stabilization and boost its degradation. To explore whether cyclin B3 degradation also contributes to the absence of Erp1 in ΔGwl oocytes, we performed a double Gwl/cyclin B3 Trim-Away experiment. We first confirmed that both proteins were effectively degraded in this experiment and subsequently measured by western blot the levels and the phosphorylation of the meiotic proteins (Fig. 5D). Interestingly, unlike c-Mos expression, the loss of cyclin B3 led to the accumulation of Erp1 upon GVBD in the absence of Gwl, a phenotype that was significantly enhanced when we supplemented c-Mos in our double Gwl/cyclin B3 Trim-Away assay. Importantly, in the absence of Gwl, c-Mos expression and Rsk activation appears not to be essential for Erp1 stabilization upon cyclin B3 degradation (Fig. 5D). These phenotypes were specific of the hyperactivation of PP2A-B55 upon Gwl depletion as they were fully rescued by microinjection in these oocytes of a thio-phosphorylated Arpp19 in the Gwl phosphorylation site S71 (Fig. 5E). Together, these data demonstrate that Gwl coordinates both, cyclin B3/CDK1 and c-Mos/MEK1/MAK1/Rsk cascade by modulating PP2A-B55 activity to confer the correct temporal pattern of Erp1 stabilization and MII arrest.

### Cyclin B3 degradation is mediated by the APC/C and conserved destruction motifs in Xenopus mitotic egg extracts

**Fig. 6:**
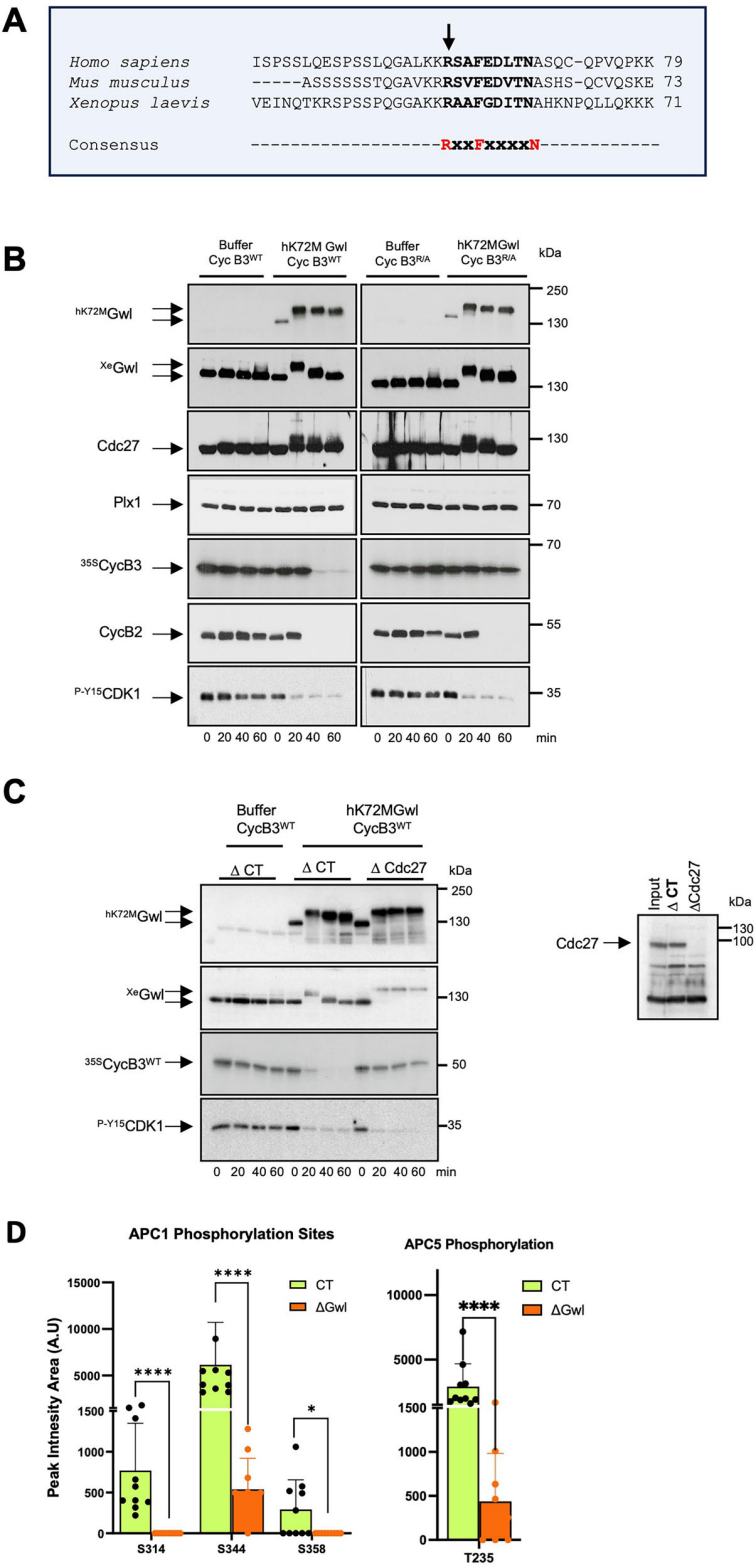
Cyclin B3 degradation is mediated by the APC/C. **A** Sequence alignment of the putative Dbox motifs of Cyclin B3 across different species. This motif is conserved in the Xenopus orthologue. **B** Interphase Xenopus egg extracts were supplemented with wildtype (WT) or mutated (R51A) in vitro translated ^35^S-Cyclin B3 protein in the presence or the absence of human Gwl K72M hyperactive kinase (hK72M). Samples were taken at indicated time-points and immunoblotted to examine human (^hK72M^Gwl) and Xenopus Gwl (^Xe^Gwl), Cdc27, Plx1, Cyclin B2, and phosphorylation of CDK1 on Y15. ^35S^Cyclin B3 was revealed by autoradiography. Data are representative of at least three different experiments **C** Interphase Xenopus egg extracts were depleted of the APC3-Cdc27 protein and supplemented with in vitro translated ^35^S-Cyclin B3 protein. Samples were then used for immunoblotting. Cyclin B3 levels were revealed by autoradiography. Complete depletion of Cdc27 in these extracts was confirmed by Western blot. Data representative for at least three different experiments. **D** Peak intensity area of the indicated phosphosites on the APC1 and APC5 subunits in control (Green) and ΔGwl (Orange) oocytes obtained by mass spectrometry in GVBD and GVBD + 4 h combined conditions. Data are expressed as mean ± Standard Deviation (SD) from ten independent replicates. Statistical analyses using two-sided non-parametric Mann–Whitney *U* test were performed. *p < 0.032; ****p < 0.0001. *n* = 5.

In order to further investigate the mechanisms by which Gwl depletion stabilizes cyclin B3, we next examined how cyclin B3 was degraded in mitotic Xenopus egg extracts. In human cells and mouse oocytes, cyclin B3 degradation takes place concomitantly with the activation of the APC/C, and the mutation of a putative Dbox motif (RXXFXXXXN) present in its amino acid sequence induces its stabilization^26,27^. This strongly suggests that the APC/C complex may be the E3 ubiquitin ligase responsible for targeting cyclin B3 degradation. Interestingly, this putative Dbox sequence is also conserved in Xenopus cyclin B3 (Fig. 6A). We thus, checked whether the mutation of this Dbox could also stabilize the protein in mitotic Xenopus egg extracts. To this, we evaluated the stability of either a wild-type or a Dbox arginine-to-alanine (R/A) mutant form of a [^35^S]-methionine-radiolabelled cyclin B3 in Xenopus egg extracts in which we induced mitotic entry by the addition of the active K72M mutant form of human Gwl (hK72MGwl) (Fig. 6B). As expected, we observed that wild-type cyclin B3 was kept unchanged in interphase extracts whereas it was degraded 20 min upon hK72MGwl addition concomitantly with the degradation of endogenous cyclin B2. However, the R/A cyclin B3 mutant remained stable. Interestingly, the APC/C subunit APC3/Cdc27 displayed a normal phosphorylation, and endogenous cyclin B2 degradation was not perturbed under these conditions, indicating that R/A mutant did not interfere with APC/C activity or with its capacity to degrade other cyclins. To further assess the role of the APC/C in cyclin B3 degradation, we next measured the stability of wild type cyclin B3 upon depletion of the APC/C subunit APC3/Cdc27 in these extracts. Confirming the role of the APC/C in cyclin B3 proteolysis, we observed its complete stabilization under these conditions (Fig. 6C).

### Greatwall modulates APC/C activation and orchestrates the temporal pattern of protein degradation during meiosis in Xenopus oocytes

**Fig. 7:**
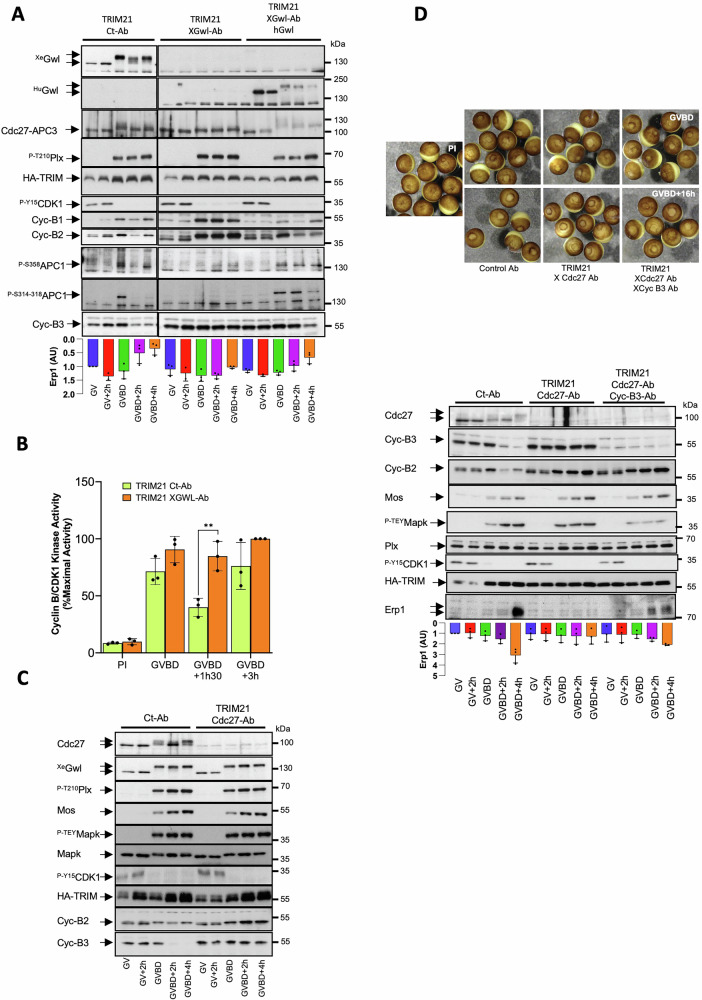
Inactive APC/C resulting from Gwl loss prevents Erp1 accumulation and cyclin B3 degradation. **A** The levels and the phosphorylation of the indicated proteins of maturing oocytes injected with HA-TRIM321 mRNA and control and XeGwl antibodies, and supplemented or not with the human Gwl protein were measured by western blot. Data representative of two different experiments. Cyclin B3 western blot signal was quantified in three different experiments and represented as mean ± SD. **B** Cyclin B/CDK1 kinase activity was measured in vitro in CDK1 immunoprecipitates from control (Green) or XeGwl (Orange) antibodies injected oocytes at the indicated time-points using H1 protein as a substrate. Values are represented as mean ± SD of the percentage of maximal activity from three different experiments. Statistical analyses using two-sided non-parametric Mann–Whitney *U* test were performed. **p < 0.005. *n* = 3. **C** Prophase I arrested oocytes were injected with HA-TRIM21 mRNA and control or anti-Cdc27 antibodies and treated with Pg. The levels and phosphorylation of the indicated proteins were analysed. Data are representative of at least three different experiments. **D** As for (**C**) except that a half of the anti-Cdc27 injected antibodies were also injected with anti-Cyclin B3 antibodies. Data representative of three different experiments. Erp1 western blot signal was quantified in the three different experiments and represented as mean ± SD. Pictures of the treated oocytes at the different time-points are shown.

Data above demonstrate that in Xenopus egg extracts, cyclin B3 is degraded by the APC/C during mitotic division. The activity of this E3 ligase is controlled by the phosphorylation of different subunits of this complex, including APC1, APC5, and APC3/Cdc27^28–30^. We thus hypothesized that the stabilization of cyclin B3 observed in Gwl-depleted maturing oocytes could result from a dephosphorylation and inactivation of the APC/C. Additionally, as supported by our proteomic findings, this inactivation would be also associated with the accumulation not only of cyclin B3, but also of cyclin B1 and B2, resulting in an increase of total cyclin/CDK activity. According to this hypothesis, our phosphoproteomic data highlighted a drastic dephosphorylation of APC1 at residues S314, S344, and S358, three essential phosphorylation sites controlling APC/C activity^30,31^, as well as an additional dephosphorylation of APC5 on T235 (Fig. 6D). To confirm these data, we examined APC/C phosphorylation by western blot analysis in control and Gwl-devoid maturing oocytes. As expected, oocytes lacking Gwl displayed a dephosphorylation of APC3/Cdc27, and of APC1 in S358 and S314-318 sites, as well as an accumulation of cyclin B1, B2, and B3 from GVBD (Fig. 7A). Conversely, in control oocytes Cdc27/APC3 and APC1 were phosphorylated, and cyclin B1, B2, and B3 were degraded from GVBD + 2 h. Because of the accumulation of cyclin B1, cyclin B2, and B3 in Gwl depleted oocytes, we also observed a significant rise of cyclin B/CDK activity upon GVBD (Fig. 7B), however, this increase was not sufficient to phosphorylate APC/C subunits due to the hyperactivation of its counterbalancing phosphatase PP2A-B55. Finally, we checked whether these modified parameters were restored when huGwl was microinjected in Gwl Trim-Away-treated oocytes. Indeed, the ectopic addition of human Gwl in these extracts fully restored the control pattern, namely, the phosphorylation of the different APC/C subunits from GBVD, the partial degradation of cyclin B1 and B2 at GVBD, its re-accumulation at MII, and the loss of cyclin B3 from GBVD + 2 h (Fig. 7A). Together, these data strongly support the role of Gwl in conferring correct activation of the APC/C and highlight its critical function in orchestrating the temporal pattern of protein degradation during meiotic progression.

### Inactive APC/C resulting from Gwl loss impairs cyclin B3 degradation and prevents Erp1 accumulation

Data above demonstrate that, during meiotic progression, the depletion of Gwl in oocytes does not allow the accumulation of Erp1 due to the absence of c-Mos and the stabilization of cyclin B3. They also demonstrate that the APC/C is not correctly activated in Gwl-devoid oocytes. Finally, we provide data showing that, as for cyclin B1 and B2, cyclin B3 is degraded via an APC/C-dependent ubiquitination. However, hitherto, we lacked direct evidence proving a causal relationship between inactive APC/C and the absence of Erp1 in these oocytes. Moreover, we have no data establishing whether inactive APC/C could modulate Erp1 levels directly, by promoting the accumulation of cyclin B3, or indirectly, by preventing c-Mos stabilization and MAPK1 activation. To obtain a definitive proof, we first inactivated APC/C in oocytes by APC3/Cdc27 Trim-Away, and we evaluated the activation of c-Mos-MAPK1 during meiosis progression. Immunoblot analysis confirmed that c-Mos was correctly accumulated and MAPK1 was fully phosphorylated in these oocytes, in which, as expected, cyclin B2 and B3 were accumulated, attesting for the inactivation of the APC/C upon APC3/Cdc27 depletion (Fig. 7C). These data prove that the APC/C does not modulate the c-Mos-MAPK1 pathway. Finally, we assessed the effect of the concomitant inactivation of the APC/C and depletion of cyclin B3 in maturing oocytes by performing a double APC3/Cdc27-cyclin B3 Trim-Away experiment. Importantly, as expected, the inactivation of the APC/C promoted cyclin B2 accumulation and the lack of Erp1, whereas the concomitant depletion of both the APC3 and cyclin B3 fully restored Erp1 accumulation in these oocytes (Fig. 7D), indicating that perturbed APC/C activity stemming from Gwl loss prevents Erp1 stabilization by blocking cyclin B3 degradation. Thus, Gwl controls Erp1 accumulation by two independent pathways: (a) the modulation of c-Mos stability, and (b) the control of the APC/C via the degradation of cyclin B3.

### Gwl depletion in maturing oocytes disrupts DNA condensation, spindle formation, and spindle positioning under the cortex

Our biochemical data above demonstrate that Gwl depletion prevents MAPK cascade activation and Erp1 stabilization, but do not provide information about the impact of these changes in oocyte meiotic progression. In order to assess the cellular consequences of Gwl depletion in maturing oocytes, we implemented immunofluorescence microscopy.

**Fig. 8:**
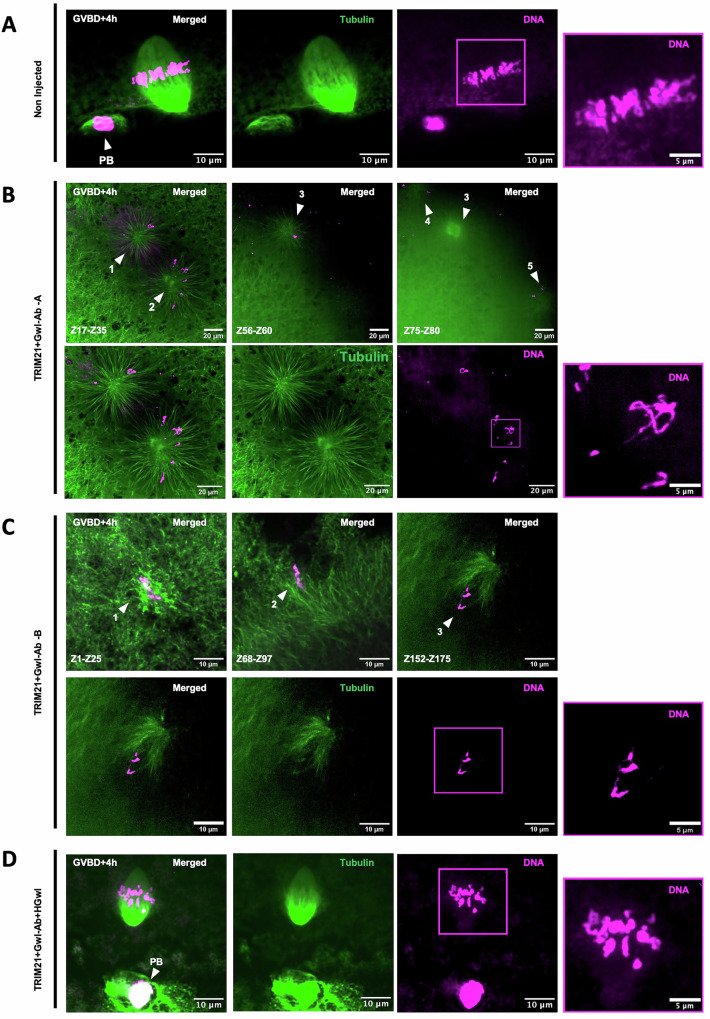
Gwl depletion in maturing oocytes disrupts DNA condensation, spindle formation, and spindle positioning under the cortex. Shown are representative images of anti-tubulin (green) and DNA (magenta) immuno-stained oocytes at 4 h upon GVBD. **A** Merged, tubulin, and DNA Z-projects of representative confocal images of non-injected oocytes. Scale Bars 10 µm and 5 µm for zoomed images. Arrowhead: Polar Body (PB) Data are representative of at least three different experiments. **B** Upper panels: Merged Z-project images of the indicated Z planes of HA-TRIM21 mRNA and XeGwl Ab injected oocytes. Arrowheads point out asters and chromosomes visible at different oocyte depths (indicated Z planes). Lower panels: merged, tubulin, and DNA Z-projects comprising all Z planes showed above. Scale Bars 20 µm and 5 µm for zoomed images. Data are representative of at least three different experiments. **C** As for (**B**) except that other HA-TRIM21 mRNA and XeGwl Ab injected oocyte was imaged. Scale Bars 10 µm and 5 µm for zoomed images. **D** Merged, tubulin, and DNA Z-projects of representative confocal images of oocytes injected with HA-TRIM21, XeGwl Abs, and huGwl protein. Scale Bars 10 µm and 5 µm for zoomed images. Arrowhead: Polar Body (PB). Data are representative of at least three different experiments.

Strikingly, Gwl depletion blocked first meiotic division since polar body was never found in ΔGwl oocytes (Fig. 8B, C) compared to controls (Fig. 8A, PB). Moreover, they lacked observable spindles, suggesting that such structures either failed to form or proved unstable. Instead, several prominent asters appeared, distributed at varying depths within the oocyte far from the cortex (see Fig. 8B, C, upper panels, asters present at three different Z plane sections). This phenotype was associated with the presence of isolated, partially condensed chromosomes (Fig. 8B, C, zoomed area) also present at different depths (Fig. 8B, C, chromosomes at different Z plane sections). Interestingly, asters were always located close to one or more chromosomes, suggesting that their formation or expansion would be dependent on the presence of chromatin. These phenotypes were specific as they were restored and polar body was extruded upon the co-injection of huGwl protein with HA-TRIM21 mRNA and Gwl Abs (Fig. 8D). Thus, Gwl is not only crucial for MAPK cascade activation and Erp1 stabilization, but it is also essential for correct spindle formation, DNA condensation, and first meiotic progression.

## Discussion

Our study identified new critical roles of Gwl and of cyclin B/CDK/PP2A-B55 balance in the control of meiotic progression in Xenopus oocytes. We used the Trim-Away approach to eliminate this kinase from prophase I-arrested Xenopus oocytes. This technique that, unlike morpholinos, directly induces an efficient and specific degradation of the targeted protein, allowed us to fully eliminate Gwl kinase from prophase I-arrested Xenopus oocytes (note that Gwl is already present in stage VI oocytes). Unexpectedly, we first find that Gwl activity was not required to activate cyclin B/CDK1 in Pg-mediated meiotic resumption. This result contrasts with previously data showing that Gwl depletion by immunoprecipitation from G2 Xenopus egg extracts blocks mitotic entry by preventing Cdc25 phosphorylation and cyclin B/CDK1 activation^9^.

While the underlying cause of these differences remains unclear, we propose that the different balance between inhibitory Myt1/Wee1 kinases and activatory Cdc25 phosphatase in prophase I oocytes (expressing Myt1 but not Wee1) and G2 Xenopus egg extracts (expressing both Myt1 and Wee1) may play a significant role.

Indeed, PP2A-B55 phosphatase prevents cyclin B/CDK1 activation by dephosphorylating Myt1, Wee1, and Cdc25, triggering the activation of the former and the inhibition of the latter. If Myt1/Wee1 activity is higher than the one of Cdc25, cyclin B/CDK1 cannot be activated. Conversely, if Cdc25 activity surpasses Myt1/Wee1 inhibitory activity, cyclin B/CDK1 will be activated. In this line, as reported above, Wee1 is not expressed in prophase I-arrested oocytes and consequently, CDK1 inhibitory activity may be lower in these oocytes^14^. Consistent with this notion, here we demonstrate that the expression of Wee1 in prophase I-arrested oocytes devoid of Gwl blocks GVBD. Moreover, we observe that the expression of Myt1 at similar levels does not prevent meiotic resumption, indicating that Myt1 has a lower cyclin B/CDK1 inhibitory activity than Wee1. Thus, down-regulation of Wee1 translation before GVBD might be a key mechanism for the oocyte to permit prophase I-release by Pg.

Although cyclin B/CDK1 is activated upon Pg addition in Gwl depleted prophase I arrested oocytes, we observed major protein phosphorylation changes during meiotic progression. Accordingly, our data showed that protein phosphorylation modifications (hyperphosphorylation or dephosphorylation) were present in 20% of the phosphoproteome. Moreover, we could establish that 40% of modified phosphosites corresponded to S-T/P cyclin B/CDK1 consensus motifs known to be directly targeted by this kinase and by the PP2A-B55 phosphatase. As expected by the preference of PP2A-B55 for TP phosphorylated sites, we identified a higher percentage of dephosphorylated TP compared to SP motifs, whereas hyperphosphorylation vs dephosphorylation percentage ratio was increased in SP sites. Interestingly, a higher percentage of dephosphorylation than hyperphosphorylation in both SP and TP motifs was present at GVBD, whereas this situation was inverted 4 h later. These differences highlight the significant effect of the hyperactivation of PP2A-B55 stemming from Gwl depletion in meiotic progression. Considering our above data, we propose that the rapid hyperactivation of PP2A-B55 upon Gwl loss would delay phosphorylation of meiotic substrates till cyclin B protein levels will provide sufficient cyclin B/CDK1 threshold activity able to overpass phosphatase activity. This delay would explain protein dephosphorylation at GVBD. However, as meiosis progresses, and due to the incapacity of the APC/C to be phosphorylated and activated in the absence of Gwl, cyclin B1, B2, and B3 accumulate, increasing total cyclin B/CDK1 activity and promoting hyperphosphorylation in late maturing oocytes (Fig. S5). Moreover, besides the direct phosphorylation/dephosphorylation of meiotic substrates by cyclin B/CDK1 and PP2A-B55, the imbalance between these two enzymes might also promote indirect effects by activating some cascades that could participate to late protein hyperphosphorylation but, also by inhibiting others that could be essential for the coordination of the different meiotic events. Accordingly, our data demonstrate that, besides abnormal high cyclin B/CDK1 activity in Gwl depleted oocytes, c-Mos is not accumulated, and MAPK/Rsk pathway remains inactive in maturing oocytes. This dysregulation, together with an increase of cyclin B3 amount, prevent the stabilization of the inhibitor of the APC/C, Erp1. Indeed, both c-Mos/MEK1/MAPK1/Rsk pathway and the cyclin B3/CDK1 kinase are essential to provide the correct temporal pattern of Erp1 protein expression during meiosis. Erp1 is dually phosphorylated by cyclin B3/CDK1 and by Rsk, with the first one inducing its degradation until anaphase I and the second one promoting its stabilization from this stage of meiosis until fertilization^4,23,27^ (Fig. 4A). Cyclin B3 is already present in prophase I-arrested oocytes and degraded upon anaphase I, when c-Mos starts to be accumulated. Thus, Erp1 is actively degraded before anaphase I and is further accumulated by Rsk-dependent phosphorylation from this stage to provide enough APC/C inhibitory activity to block oocytes in metaphase II.

We show that improper PP2A-B55 hyperactivation induced by Gwl loss promotes c-Mos degradation and prevents the activation of MAPK/Rsk. Although we do not know the exact mechanism involved in c-Mos proteolysis upon Gwl depletion, we showed in the past that CDK1-dependent phosphorylation of c-Mos on S3 protects this protein from ubiquitin-mediated degradation^32^. In this scenario, increased PP2A-B55 activity may remove this stabilizing mark, making c-Mos susceptible to proteasomal degradation.

Our data identified a primary cascade responsible for cyclin B3 stabilization upon Gwl depletion in maturing oocytes. Together, our phosphoproteomic and biochemical data demonstrate that in Gwl-devoid oocytes, APC1, APC5, and APC3/Cdc27 remain dephosphorylated during meiotic progression, preventing the activation of this E3 ligase and impairing cyclin B1, B2, and B3 degradation at the MI-AI transition. As recently reported in mouse oocytes^27^, we identified cyclin B3 as a substrate of APC/C in Xenopus oocytes. More importantly, we also demonstrate that Gwl is essential for the activation of the APC/C and the degradation of cyclin B3, allowing the stabilisation of Erp1 crucial for the establishment of metaphase II arrest. Accordingly, when cyclin B3 was experimentally depleted in Gwl-deficient oocytes, Erp1 was restored, demonstrating that APC/C-mediated degradation of cyclin B3 plays a crucial role in maintaining Erp1 stability in these oocytes. These results reveal a feedback loop that is tightly regulated by Gwl/PP2A-B55. In this loop, the APC/C complex controls its own inhibitor, Erp1, by degrading its substrate, cyclin B3. Interestingly, although previous data showed that the stabilization of Erp1 at metaphase I upon cyclin B3 depletion requires c-Mos^4^, we demonstrate that this protein is not required in the absence of Gwl. In this regard, it has been shown that the presence of c-Mos during meiotic maturation promotes Rsk-dependent phosphorylation of Erp1, triggering its binding to PP2A and promoting the dephosphorylation of cyclin B/CDK1-dependent phosphosites that prime this protein for SCF-βTrcp-mediated ubiquitination and degradation^5^. Thus, in the absence of Gwl (e.g., in the presence of a hyperactive PP2A-B55), cyclin B/CDK1 phosphosites in Erp1 might be constantly dephosphorylated, making Rsk activity dispensable for its stabilisation.

Interestingly, our immunofluorescence data revealed that, although able to activate cyclin B/CDK1 and to perform GVBD, Gwl-depleted oocytes failed to assemble proper bipolar spindles or complete chromosomes condensation, preventing first polar body extrusion. Instead, they displayed prominent asters always close to partially condensed chromosomes. Since ΔGwl-dependent hyperactivation of PP2A-B55 triggers widespread protein dephosphorylation, it is likely that this phenotype arises from multiple pleiotropic defects rather than a single factor. One of these factors could be MAPK inactivation, whose its inhibition in Xenopus egg extracts has been shown to induce the disassembly of the bipolar spindle into large asters^33^. Additionally, our phosphoproteome revealed a strong dephosphorylation of Xenopus Ncaph.S (the orthologue of human CAP-H2) at the Plk1 target site (S224) essential for condensin activation^34^. This dephosphorylation impairs condensin I recruitment and ATPase-driven DNA compaction and thus might additionally contribute to chromosome undercondensation. Finally, we additionally identified a drastic dephosphorylation of key actin cytoskeleton regulators (WASF, PXN, ARHGAP) playing a prominent role in cortical spindle migration^35^ that could explain the scattered distribution of asters and chromosomes in the oocytes.

In summary, our data demonstrate that Gwl ensures the coordination of meiotic events by maintaining the proper balance between cyclin B/CDK1 kinase and PP2A-B55 phosphatase activities. This is achieved through the accurate phosphorylation/activation of the APC/C, the accumulation of c-Mos and Erp1, and the temporal phosphorylation of proteins involved in microtubule dynamics, chromosome condensation, and actin cytoskeleton essential for meiosis I-II progression.

## Methods

### Experimental model and subject details

#### Rabbits husbandry

The rabbits were obtained from Charles River Laboratories France and kept in a research facility for rabbits at UMR IRD 224-CNRS 5290, University of Montpellier (facility and authorisation approved by the French government). Approval numbers I3417221 and 2024012616358373 v5, respectively).

#### *Xenopus laevis* induction and husbandry

Regulations for the use of *Xenopus laevis*, as outlined in the Animals Scientific Procedures Act (ASPA) and implemented by the Direction Generale de la Recherche et Innovation, Ministère de l’Enseignement Supérieur de l’Innovation of France, were followed.

Frogs were obtained from «Aquatic facilities: TEFOR Paris-Saclay», France, and kept in a *Xenopus* research facility at the CRBM (Facility Center approved by the French Government. Approval n° C34-172-39. Authorisation approval number APAFIS #40182-202301031124273 v4).

Females were injected of 500 U Chorulon (Human Chorionic Gonadotrophin), and oocytes layed 18 h later were used for experiments. Adult females were exclusively used to obtain eggs. All procedures were approved by the Direction Generale de la Recherche et Innovation, Ministère de l’Enseignement Supérieur de la l’Innovation of France (Approval n° APAFIS#40182-202301031124273v4).

#### Preparation of interphase Xenopus egg extracts

Adult female Xenopus laevis were injected with 500 U of human chorionic gonadotropin (Chorulon) to induce ovulation. Eggs were collected approximately 18 h post-injection in XB buffer (100 mM KCl, 1 mM MgCl_2_, 0.1 mM CaCl_2_, 50 mM sucrose, 10 mM HEPES, pH 7.8) at room temperature. Selected oocytes were dejellied using 2% cysteine solution (pH 7.8) for 5–7 min with gentle agitation. Metaphase II laid oocytes were treated with calcium ionophore A23187 (final concentration of 2 μg/ml) to induce meiotic exit and collected 35 min later. Extracts were prepared by centrifugation at 10,000 × *g* for 30 min at 4 °C. The cytoplasmic fraction was collected, supplemented with protease inhibitors, and centrifuged again at 10,000 × *g* for 30 min at 4 °C. Supernatant was finally collected, aliquoted, and frozen in liquid nitrogen for storage at −80 °C.

#### Plasmids

Xenopus full length cyclin B3 cDNA was amplified from Xenopus ovary cDNAs, cloned blunt into EcoRV site of the pCS2 vector, and subsequently subcloned either in the EcoRI-XbaI sites of pMalP2 or in the EcoRI-XhoI sites of pGEX4T2.

Full length human TRIM21 cDNA was cloned into the EcoRV cloning site of the HA-pCS2 vector. Full length Xenopus Myt1 and Wee1 cDNAs from Xenopus ovary cDNAs were cloned into the EcoRV cloning site of the HA-pCS2 vector.

Xenopus full length c-Mos was amplified from Xenopus ovary cDNAs and subcloned blunt into the EcoRV site of the pCS2 vector. This vector was subsequently used to subclone this sequence in EcoRI-NotI cloning site of pGEX4T1.

#### Site-Directed Mutagenesis

Point mutation of Xenopus cyclin B3 (R51A) was introduced by PCR in pCS2-Cyclin B3 plasmid using the Pfu ultra II fusion DNA polymerase (Agilent). Mutation was verified by DNA sequencing. Oligonucleotides used for site directed mutagenesis were purchased from Eurogentec.

#### mRNA preparation for Xenopus oocyte injection and in vitro translation

For expression of TRIM21, Wee1, or Myt1, corresponding pCS2 vectors were used to obtain the mRNAs using the mMESSAGE mMACHINE SP6 RNA polymerase Ultra Kit (Ambion) according to the manufacturer’s instructions.

Wild type cyclin B3 and R51A mutant mRNAs were transcribed from pCS2-Cyclin B3 (Wt and R51A) vectors with the mMessage mMachine SP6 RNA polymerase kit (Ambion) and translated in vitro using reticulocyte lysate system (Promega).

The pCS2 vector coding for the 5’ and 3’ UTR regions of c-Mos and the coding region for the GFP protein was synthesized by GeneCust. Sequence is available in Supplementary Information.

#### Antibody production and purification, and immunoblots

MBP or GST fusion cyclin B3 proteins were expressed in *E. Coli*. For the immunization protocol, GST-Cyclin B3 was purified from inclusion bodies, subjected to SDS–PAGE and electroeluted according to standard procedures. This protein was next dialysed against 50 mM NaCl, 50 mM NaHCO_3_ buffer, and used to immunize rabbits. Soluble MBP-Cyclin B3 was purified on an amylose column and coupled to CNBr-sepharose beads for affinity purification of immune sera^36^.

GST-fused Xenopus c-Mos protein was expressed in *E. Coli* and purified using a gluthatione column. Purified c-Mos was then dialyzed against XB buffer, aliquoted, and stored at −80 °C.

For western blot, 1 µl of interphase or maturing oocyte extracts was loaded on a polyacrylamide gel and transferred onto Immobilon-P membrane that was next blocked with 5% milk-TBST, or with 5% BSA-TBST when phospho-antibodies were used, and incubated overnight with primary antibodies in 2% milk-TBST. HRP-conjugated secondary antibodies directed against rabbit (Cell Signaling) or mouse (Santa Cruz Biotechnologies) were incubated for 30 min at RT in 2% milk-TBST.

#### TRIM-Away in Xenopus oocytes

Prophase I-arrested oocytes were harvested after surgery, washed in OR-2 buffer (82.5 mM NaCl, 2 mM KCl, 1 mM MgCl_2_, 5 mM HEPES, pH 7.2), and incubated with 1 mg/ml of collagenase. After dissociation, oocytes were selected, washed again in OR-2 buffer, and injected with 20 nl of a mixture containing 10 ng of mRNA TRIM21 and 40 ng of control (anti-GST) or affinity purified (Xenopus Gwl, cyclin B3, or Cdc27) antibodies. For complementation studies, 20 ng of human wildtype Gwl and/or 40 ng of Xenopus c-Mos protein were subsequently injected. Upon injection, oocytes were incubated overnight at 19 °C in NDS medium (96 mM NaCl, 2 mM KCl, 1.8 mM CaCl_2_, 1 mM MgCl_2_, 5 mM HEPES, 2.5 mM Pyruvate, 0.05 mM Gentamycine) and the day after, progesterone was added to a final concentration of 37 µM in Merriam buffer (10 mM HEPES, 0.82 mM MgSO_4_, 88 mM NaCl, 0.33 mM Ca(NO_3_)2, 1 mM KCl and 0.41 mM CaCl_2_, pH7.4). Oocytes in prophase I, 2 h upon progesterone addition, at GVBD (assessed by pigment rearrangement in the animal pole), and 4 h after GVBD were recovered. Collected oocytes were then homogenized in extraction buffer (80 mM β-glycerophosphate, 20 mM EGTA, 15 mM MgCl_2_, and 2 mM EDTA), centrifuged at 15,000 × *g* for 10 min at 4 °C, and lysates used for Western blotting.

#### Immunoprecipitation

For immunoprecipitation, 2 μg of anti-CDK1 or anti-Rsk antibodies were incubated with 20 μl Dynabeads protein G (Invitrogen) for 30 min and, after washing with XB buffer, incubated again with 80 μl of the lysate corresponding to four oocytes for an additional period of 30 min. Beads were then washed to eliminate non-specific bound proteins and used for kinase activity assays.

For immunodepletion, 10 μg of affinity-purified antibodies against APC3-Cdc27 or GST (Glutathione S-Transferase) were bound for 30 min to 20 μl Dynabeads protein G (Invitrogen), and then, after washing with XB buffer, added to 20 μl of Interphase egg extracts for 30 min at 20 °C. Two consecutive immunoprecipitations were performed to completely remove endogenous Cdc27 protein. Then, beads were removed, and the supernatant was incubated with radiolabelled cyclin B3 at 20 °C. Samples were taken every 20 min up to 60 min, mixed 1:9 with 2x Laemmli buffer and heated 5 min at 94 °C to be subsequently used for immunoblot analysis.

#### Kinase activity assays

CDK1 kinase activity was measured using histone 1 as a substrate. Shortly, four-oocyte CDK1 immunoprecipitates previously obtained and frozen were thawed by the addition of 15 μl of histone buffer (20 mM Hepes pH 7.2, 5 mM MgCl_2_, 100 µM ATP, and 1 mg/ml Histone 1), including 0.2μCi of [γ33P] ATP, and incubated for 10 min at room temperature. Reactions were stopped by adding 2x Laemmli sample buffer followed by heat denaturation at 94 °C for 5 min and analysed by SDS-PAGE and autoradiography.

For Rsk kinase activity measurements, Rsk1/2 immunoprecipitates from 4 oocytes were mixed with a buffer containing 1 mg/ml of dephosphorylated Kemtide peptide, 0.3 μM [γ33P] ATP, 2 mM MgCl_2_, and 50 mM Tris-HCL pH7.5 and incubated for 20 min at room temperature. Each reaction mixture was spotted onto P81 phosphocellulose paper squares (1 × 1 cm), dried for 1–2 min and washed 5 times (5 min each) with 75 mM phosphoric acid with gentle agitation to remove unincorporated [γ-³³P] ATP. A subsequent wash with acetone was performed, and papers were allowed to dry completely. Dried papers were then placed in scintillation vials, scintillation liquid was added, and ³³P incorporation was measured using a scintillation counter.

#### Arpp19 thio-phosphorylation

His-Xenopus Arpp19 (isoform 1, accession number XM018251008.1)^37^ were produced in *Escherichia coli* and purified using TALON Superflow Metal Affinity Resin. Phosphorylation of Arpp19 on S71 by Gwl was induced by using GST-K72M hyperactive mutant form of this kinase purified from SF9 cells. For *“*in vitro*”* phosphorylation reaction, 6His-Arpp19 protein and GST-K72M Gwl kinase were mixed at a final concentration of 0.5 μg/μl and 45 ng/μl, respectively, in a reaction buffer (1 mM ATP^γs^, 2 mM MgCl_2_, and Tris 50 mM) and incubated for 1 h.

#### Degradation assay

For protein degradation assays, 2 μl of either ^35^S-labelled WT cyclin B3 or mutant (R51A) translated proteins were incubated at room temperature with 20 μl of interphase extracts supplemented or not with HuGwl K72M protein.

#### Incubation and meiotic induction

Injected oocytes were incubated overnight at 19 °C in NDS medium. Upon overnight incubation, oocytes were transferred to Merriam Buffer supplemented with progesterone (37 μM) to induce meiotic resumption.

#### Immunofluorescence in Xenopus oocytes

For IF staining, oocytes were transferred to a fixation solution (100% methanol) and incubated overnight with slow shaking at 4 °C. Oocytes were then re-hydrated with methanol 70% and 30% for 20 min, successive incubations, washed for 20 min in 0.1% Triton X-100 in PBS (PBST), and blocked for 45 min in PBST + 2% BSA. Next, oocytes were incubated with primary anti-tubulin antibody (1/500) in PBST + 2% BSA for 72 h at 4 °C in the dark and, after three washes of 20 min in TBST + 0.1% BSA, treated with secondary Alexa-Fluor^TM^ 546- Goat anti-Rat antibody (1/1000) at 4 °C for 48 h in TBST + 2% BSA. Upon three supplementary washes of 20 min in PBST + 0.1% BSA, DNA was stained by treating the oocytes with SYTOX (1/1000) for 90 min at room temperature in the dark. Oocytes were next submitted to three more washes of 20 min with PBS, dehydrated using consecutive incubations with 30, 60, and 100% methanol in PBS for 30 min, and subsequently incubated twice for 1 min in benzoate and benzyl alcohol (ratio 1:2) to induce transparency. Oocytes were finally mounted in the same media on a 35 mm Glass bottom dish with micro-well cover glass (Cellvis). Oocytes were gently rotated using a tweezer to place the spindle closer from the cover glass on glass slides and imaged using an Olympus inverted microscope equipped with a spinning disk Yokogawa W1 confocal device and coupled to sCMOS Fusion BT camera (Hamamatsu). Images were acquired using a 60x UPLSAPO 1.3 NA silicone immersion objective.

#### Sample preparation prior to LC-MS/MS analysis

Ten oocytes per condition were homogenized in extraction buffer, centrifuged at 15,000 × *g* for 10 min at 4 °C, and supernatants recovered. A six-time volume of cold acetone (−20 °C) was then added to the samples containing about 250 µg of protein extracts. Vortexed tubes were incubated overnight at −20 °C, then centrifuged for 10 min at 12,900 × *g* at 4 °C. Supernatant was removed, then the protein pellets were dissolved in 8 M Urea- 25 mM NH_4_HCO_3_ buffer. Samples were then reduced with 10 mM TCEP-HCl and alkylated with 20 mM MMTS. After a 16-fold dilution in NH_4_HCO_3_, samples were then digested overnight at 37 °C by a mixture of trypsin/Lys C (1/100 Enzyme/Substrate ratio). The digested peptides were desalted on Sep-Pak Classic C18 cartridges. Cartridges were sequentially washed with 2 ml methanol, 2 ml of a 70% (v/v) aqueous ACN containing 1% (v/v) TFA, and equilibrated with 2 ml of 1% (v/v) aqueous TFA. The digested peptides were acidified with 1% (v/v) aqueous TFA, applied to the column, and washed with 2 × 1 ml of a 1% (v/v) aqueous FA. Peptides were eluted by applying 2 × 500 µL of a 70% (v/v) aqueous ACN containing 0.1% (v/v) FA. Eluates were vacuum-dried. Phosphopeptide enrichment was then performed according to manufacturer’s procedure, starting from the lyophilized peptide samples. Eluted phosphopeptides and peptides from total proteome were loaded and desalted on Evotips provided by Evosep (Odense, Denmark) according to manufacturer’s procedure before LC-MS/MS analysis. The flow-through material resulting from the enrichment procedure was processed as well for total proteome analysis.

#### LC-MS/MS acquisition

Samples were analyzed on a timsTOF Pro 2 mass spectrometer (Bruker Daltonics, Bremen, Germany) coupled to an Evosep one system (Evosep, Odense, Denmark) operating with the 30SPD method developed by the manufacturer. Briefly, the method is based on a 44-min gradient and a total cycle time of 48 min with a C18 analytical column (0.15 × 150 mm, 1.9 µm beads, ref EV-1106) equilibrated at 40 °C and operated at a flow rate of 500 nl/min. H_2_O/0.1 % FA was used as solvent A and ACN/ 0.1 % FA as solvent B. For the phosphopeptide analysis, the timsTOF Pro 2 was operated in DDA-PASEF mode with a cycle time of 1.3 s. Mass spectra for MS and MS/MS scans were recorded between 100 and 1700 *m/z*. The total proteome was also analyzed on the instrument using a DIA-PASEF method consisting of 12 pydiAID frames with 3 mass windows per frame, resulting in a cycle time of 0.975 s as described in the Bruker Application Note LCMS 218. Collisional energy was ramped stepwise as a function of ion mobility.

#### Data analysis

MS raw files for phosphopeptide analysis were processed using PEAKS Online 11 (build 1.9, Bioinformatics Solutions Inc.). Data were searched against the SwissProt+TrEMBL Xenopus Laevis entries (downloaded January 2024, 112365 entries). Parent mass tolerance was set to 20 ppm, with fragment mass tolerance to 0.05 Da. Specific tryptic cleavages were selected, and a maximum of 2 missed cleavages was allowed. The following post-translational modifications were considered for identification: Phosphorylation (STY), Oxidation (M), Deamidation (NQ), and Acetylation (Protein N-term) as variable and Beta-methylthiolation (C) as fixed. Identifications were filtered based on a 1% FDR (False Discovery Rate) threshold at both peptide and protein group levels. Label-free quantification was performed using the PEAKS Online 11 quantification module, allowing a mass tolerance of 5 ppm, a CCS error tolerance of 0.01, and a 0.25-min retention time shift tolerance for match between runs. PEAKS Studio software was also used to format sequences with the modified amino acid flanked by ±10 amino acids.

DIA raw files for total proteome were processed using Spectronaut 18 (Biognosys, Switzerland). Data were searched against the same database. Specific tryptic cleavages were selected, and a maximum of 2 missed cleavages was allowed. The following post-translational modifications were considered for identification: Acetyl (Protein N-term), Oxidation (M), as variable, and MMTS (C) as fixed. The maximum number of variable modifications was set to 5. Identifications were filtered based on a 1% precursor and protein *Q*-value cutoff threshold. The protein LFQ method was set to automatic, and the quantity was set at the MS2 level with a cross-run normalization applied.

Multivariate statistics on protein or peptide measurements were performed using Qlucore Omics Explorer 3.9 (Qlucore AB, Lund, *SWEDEN*). A positive threshold value of 1 was specified to enable a log2 transformation of abundance data for normalization, i.e., all abundance data values below the threshold will be replaced by 1 before transformation. The transformed data were finally used for statistical analysis, i.e., evaluation of differentially present proteins or peptides between two groups using a Student’s bilateral *t*-test. Since the observed effects of Gwl kinase on phosphorylation are very close in the early and late conditions, we also statistically processed the samples considering only two Ctrl and Gwl groups of 10 replicates each (GVBD plus MII) and substracting the variance related to the timing parameter. A *p*-value better than 0.05 was finally used to filter differential candidates.

#### Phosphoproteomics data processing and filtering

Phosphoproteomic data were obtained from the file 2402007_ptmprofile_LFQ_processed doublons_annotations_Ttest CTRL vs Gwl-EF Timing-1.xlsx, which was processed in R (v4.3) using the packages {readxl}, {dplyr}, {purrr}, {stringr}, {magrittr}, {openxlsx}, {ggplot2}, {ggfortify}, {ggsci} and {ggrepel}. The data was imported with read_xlsx, and phosphorylation sites were annotated using columns for peptide sequences, protein IDs, gene names, amino acid type, and site position.

#### Phosphosite filtering and clustering

To group phosphorylation sites by protein, gene names were cleaned to remove isoform suffixes, and unique clusters were assigned to each protein (one cluster per unique gene). Sites were filtered to retain only unique phosphorylation sites per protein for each amino acid type: serine (S), threonine (T), and tyrosine (Y). This ensures that each phosphosite is only counted once per protein.

#### Analysis of variance (ANOVA) and multiple testing correction

For each retained peptide, an ANOVA was performed to assess differences in phosphorylation levels across the four experimental conditions. Resulting p-values were adjusted for multiple testing using the false discovery rate (FDR) method (Benjamini–Hochberg). Additionally, *q*-values were computed using the qvalue R package to estimate the proportion of false positives (Storey and Tibshirani, 2003).

#### Principal component analysis (PCA)

To explore the global variation in phosphorylation profiles, PCA was performed using the log2-transformed intensities of peptides significantly modulated across conditions (*q* < 0.05). Peptides were considered significantly modulated if they exhibited evidence of differential phosphorylation based on ANOVA followed by FDR correction. Statistical computing and graphics were performed using R software (https://www.R-project.org/).

The PCA was performed on a matrix where rows represent samples (biological replicates), and columns represent significantly modulated phosphopeptides. Principal components were calculated using the prcomp function with centering and scaling applied to each peptide.

The PCA scores for each sample were visualized using ggplot2 with colour coding to indicate experimental condition. Sample labels were added using ggrepel to minimize overlap. Axes were annotated with the percentage of variance explained by each principal component.

#### Heat map

Heat map was performed using the ComplexHeatmap package (Gu and Collegues, 2016).

### Quantification and statistical analysis

Differences of phosphorylation in phosphorylation levels across the different conditions in label-free mass spectrometry analysis were performed using ANOVA followed FDR correction. When PCA was performed combining GVBD and GVBD + 4H groups in control and ΔGwl conditions, a two-group *t*-test with FDR correction was used. Statistical differences in peak intensity area between GVBD and GVBD + 4H of phosphopeptides described in Figs. 4A and 6D were determined by using a two-sided non-parametric Mann–Whitney *U* test.

### Reporting summary

Further information on research design is available in the Nature Portfolio Reporting Summary linked to this article.

## Supplementary information

Supplementary Information

Reporting Summary

Transparent Peer Review file

## Source data

Source data

## Data Availability

Further information and requests for resources and reagents should be directed to and will be fulfilled by the lead contact thierry.lorca@crbm.cnrs.fr. The remaining data supporting the findings of this study, supplementary information, and Source data file are provided with this paper. The immunofluorescence images generated in this study have been deposited to Zenodo (10.5281/zenodo.19201904). The raw mass spectrometry proteomics data generated in this study have been deposited in the ProteomeXchange Consortium via the PRIDE partner repository with the dataset identifier PXD068132. Source data are provided with this paper.

## References

[1] Pei, Z., Deng, K., Xu, C. & Zhang, S. The molecular regulatory mechanisms of meiotic arrest and resumption in Oocyte development and maturation. *Reprod. Biol. Endocrinol.* **21**, 90 (2023). 37784186 10.1186/s12958-023-01143-0 PMC10544615

[2] Ohe, M., Inoue, D., Kanemori, Y. & Sagata, N. Erp1/Emi2 is essential for the meiosis I to meiosis II transition in Xenopus oocytes. *Dev. Biol.* **303**, 157–164 (2007). 17141208 10.1016/j.ydbio.2006.10.044

[3] Schunk, R. et al. A phosphate-binding pocket in cyclin B3 is essential for XErp1/Emi2 degradation in meiosis I. *EMBO Rep.* **26**, 768–790 (2025). 39747666 10.1038/s44319-024-00347-8 PMC11811201

[4] Bouftas, N. et al. Cyclin B3 implements timely vertebrate oocyte arrest for fertilization. *Dev. Cell* **57**, 2305–2320.e6 (2022). 36182686 10.1016/j.devcel.2022.09.005

[5] Wu, J. Q. et al. Control of Emi2 activity and stability through Mos-mediated recruitment of PP2A. *Proc. Natl. Acad. Sci. USA*. **104**, 16564–16569 (2007). 17881560 10.1073/pnas.0707537104 PMC2034268

[6] Castro, A. & Lorca, T. Greatwall kinase at a glance. *J. Cell Sci.* **131**, jcs222364 (2018). 30355803 10.1242/jcs.222364

[7] Lorca, T. et al. Constant regulation of both the MPF amplification loop and the Greatwall-PP2A pathway is required for metaphase II arrest and correct entry into the first embryonic cell cycle. *J. Cell Sci.* **123**, 2281–2291 (2010). 20554897 10.1242/jcs.064527

[8] Zhao, Y. et al. Roles of Greatwall kinase in the regulation of cdc25 phosphatase. *Mol. Biol. Cell* **19**, 1317–1327 (2008). 18199678 10.1091/mbc.E07-11-1099 PMC2291418

[9] Yu, J., Zhao, Y., Li, Z., Galas, S. & Goldberg, M. L. Greatwall kinase participates in the Cdc2 autoregulatory loop in Xenopus egg extracts. *Mol. Cell* **22**, 83–91 (2006). 16600872 10.1016/j.molcel.2006.02.022

[10] Mochida, S., Maslen, S. L., Skehel, M. & Hunt, T. Greatwall phosphorylates an inhibitor of protein phosphatase 2A that is essential for mitosis. *Science* **330**, 1670–1673 (2010). 21164013 10.1126/science.1195689

[11] Gharbi-Ayachi, A. et al. The substrate of Greatwall kinase, Arpp19, controls mitosis by inhibiting protein phosphatase 2A. *Science* **330**, 1673–1677 (2010). 21164014 10.1126/science.1197048

[12] Clift, D., So, C., McEwan, W. A., James, L. C. & Schuh, M. Acute and rapid degradation of endogenous proteins by Trim-Away. *Nat. Protoc.* **13**, 2149–2175 (2018). 30250286 10.1038/s41596-018-0028-3

[13] Clift, D. et al. A method for the acute and rapid degradation of endogenous proteins. *Cell* **171**, 1692–1706.e18 (2017). 29153837 10.1016/j.cell.2017.10.033 PMC5733393

[14] Nakajo, N. et al. Absence of Wee1 ensures the meiotic cell cycle in Xenopus oocytes. *Genes Dev.* **14**, 328–338 (2000). 10673504 PMC316360

[15] Kruse, T. et al. Substrate recognition principles for the PP2A-B55 protein phosphatase. *Sci. Adv.* **10**, eadp5491 (2024). 39356758 10.1126/sciadv.adp5491 PMC11446282

[16] Swartz, S. Z. et al. Selective dephosphorylation by PP2A-B55 directs the meiosis I-meiosis II transition in oocytes. *eLife* **10**, e70588 (2021). 34342579 10.7554/eLife.70588 PMC8370769

[17] Colaert, N., Helsens, K., Martens, L., Vandekerckhove, J. & Gevaert, K. Improved visualization of protein consensus sequences by iceLogo. *Nat. Methods* **6**, 786–787 (2009). 19876014 10.1038/nmeth1109-786

[18] Fedoryshchak, R. O. et al. Molecular basis for substrate specificity of the Phactr1/PP1 phosphatase holoenzyme. *eLife* **9**, e61509 (2020). 32975518 10.7554/eLife.61509 PMC7599070

[19] Ilinykh, P. A. et al. Role of protein phosphatase 1 in dephosphorylation of Ebola virus VP30 protein and its targeting for the inhibition of viral transcription. *J. Biol. Chem.* **289**, 22723–22738 (2014). 24936058 10.1074/jbc.M114.575050 PMC4132779

[20] Al-Rawi, A., Kaye, E., Korolchuk, S., Endicott, J. A. & Ly, T. Cyclin A and Cks1 promote kinase consensus switching to non-proline-directed CDK1 phosphorylation. *Cell Rep.* **42**, 112139 (2023). 36840943 10.1016/j.celrep.2023.112139

[21] Szklarczyk, D. et al. The STRING database in 2023: protein-protein association networks and functional enrichment analyses for any sequenced genome of interest. *Nucleic Acids Res.* **51**, D638–D646 (2023). 36370105 10.1093/nar/gkac1000 PMC9825434

[22] Avilov, I. et al. Phosphoproteomic identification of Mos–MAPK targets in meiotic cell cycle and asymmetric oocyte divisions. *J. Cell Biol.* **224**, e202312140 (2025). 41123450 10.1083/jcb.202312140 PMC12542822

[23] Inoue, D., Ohe, M., Kanemori, Y., Nobui, T. & Sagata, N. A direct link of the Mos-MAPK pathway to Erp1/Emi2 in meiotic arrest of Xenopus laevis eggs. *Nature* **446**, 1100–1104 (2007). 17410130 10.1038/nature05688

[24] Sun, H. et al. The global phosphorylation landscape of mouse oocytes during meiotic maturation. *EMBO J.* **43**, 4752–4785 (2024). 39256562 10.1038/s44318-024-00222-1 PMC11480333

[25] Lai, S. & Pelech, S. Regulatory roles of conserved phosphorylation sites in the activation T-loop of the MAP kinase ERK1. *Mol. Biol. Cell* **27**, 1040–1050 (2016). 26823016 10.1091/mbc.E15-07-0527 PMC4791125

[26] Nguyen, T. B. et al. Characterization and expression of mammalian cyclin B3, a prepachytene meiotic cyclin. *J. Biol. Chem.* **277**, 41960–41969 (2002). 12185076 10.1074/jbc.M203951200

[27] Karasu, M. E., Bouftas, N., Keeney, S. & Wassmann, K. Cyclin B3 promotes anaphase I onset in oocyte meiosis. *J. Cell Biol.* **218**, 1265–1281 (2019). 30723090 10.1083/jcb.201808091 PMC6446836

[28] Bansal, S. & Tiwari, S. Mechanisms for the temporal regulation of substrate ubiquitination by the anaphase-promoting complex/cyclosome. *Cell Div.* **14**, 14 (2019). 31889987 10.1186/s13008-019-0057-5 PMC6927175

[29] Chia, K. H. et al. CDK1-PP2A-B55 interplay ensures cell cycle oscillation via Apc1-loop300. *Cell Rep.* **43**, 114155 (2024). 38678563 10.1016/j.celrep.2024.114155

[30] Fujimitsu, K. & Yamano, H. Dynamic regulation of mitotic ubiquitin ligase APC/C by coordinated Plx1 kinase and PP2A phosphatase action on a flexible Apc1 loop. *EMBO J.* **40**, e107516 (2021). 34291488 10.15252/embj.2020107516 PMC8441438

[31] Fujimitsu, K., Grimaldi, M. & Yamano, H. Cyclin-dependent kinase 1-dependent activation of APC/C ubiquitin ligase. *Science* **352**, 1121–1124 (2016). 27103671 10.1126/science.aad3925

[32] Castro, A. et al. Cyclin B/cdc2 induces c-Mos stability by direct phosphorylation in Xenopus oocytes. *Mol. Biol. Cell* **12**, 2660–2671 (2001). 11553706 10.1091/mbc.12.9.2660 PMC59702

[33] Horne, M. M. & Guadagno, T. M. A requirement for MAP kinase in the assembly and maintenance of the mitotic spindle. *J. Cell Biol.* **161**, 1021–1028 (2003). 12821640 10.1083/jcb.200304144 PMC2172988

[34] Kagami, Y., Ono, M. & Yoshida, K. Plk1 phosphorylation of CAP-H2 triggers chromosome condensation by condensin II at the early phase of mitosis. *Sci. Rep.* **7**, 5583 (2017). 28717250 10.1038/s41598-017-05986-7 PMC5514044

[35] Yi, K. et al. Dynamic maintenance of asymmetric meiotic spindle position through Arp2/3 complex-driven cytoplasmic streaming in mouse oocytes. *Nat. Cell Biol.* **13**, 1252–1258 (2011). 21874009 10.1038/ncb2320 PMC3523671

[36] Lorca, T. et al. Fizzy is required for activation of the APC/cyclosome in Xenopus egg extracts. *EMBO J*. **17**, 3565–3575 (1998). 10.1093/emboj/17.13.3565 PMC1170693 9649427

[37] Ma, S. et al. Greatwall dephosphorylation and inactivation upon mitotic exit is triggered by PP1. *J. Cell. Sci.* **129**, 1329–1339 (2016). 26906418 10.1242/jcs.178855

