# Supplementary material for: Greatwall depletion from Xenopus oocytes reveals a key role of the cyclin B/CDK1-PP2A-B55 balance in the coordination of meiotic events
Source: Nat Commun. 2026 May 13;17:6361. doi: 10.1038/s41467-026-73011-5 (PMC13376403; doi:10.1038/s41467-026-73011-5)
Supplement: Supplementary file 1 — Supplementary Information [file 41467_2026_73011_MOESM1_ESM.pdf]

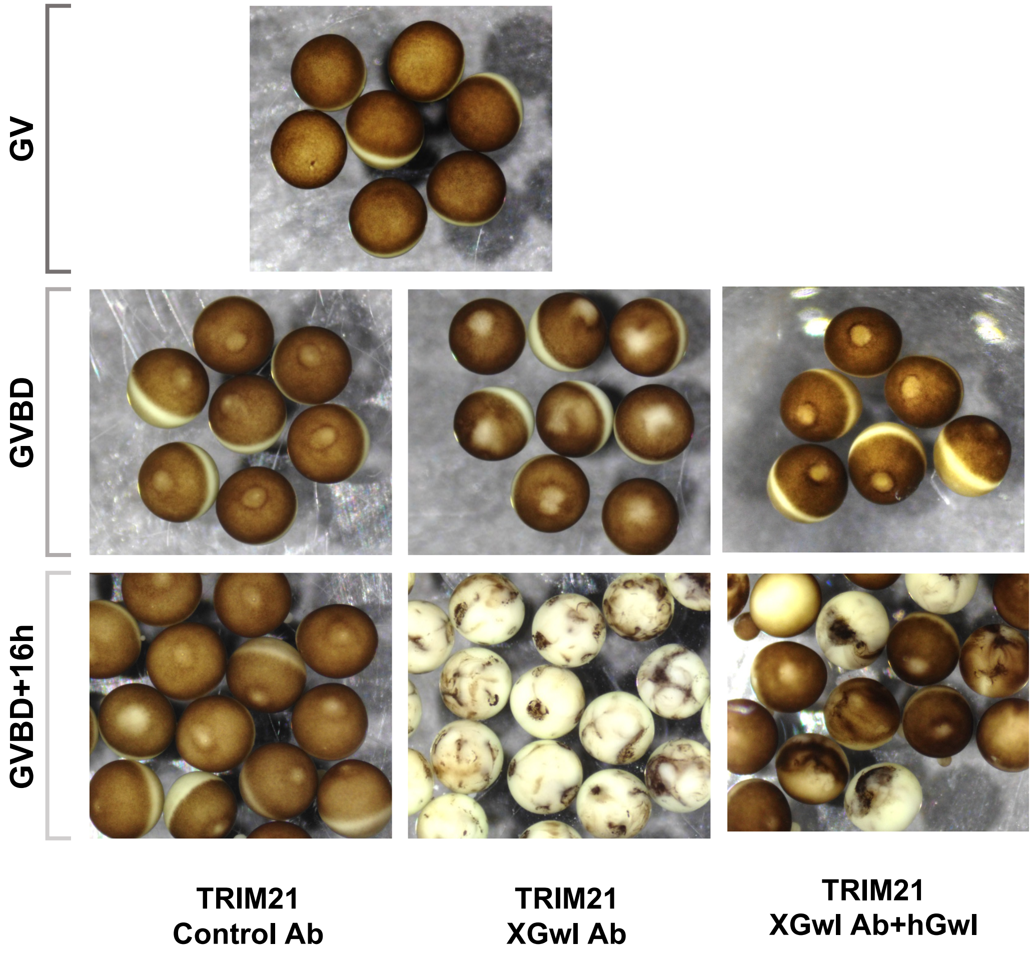

Supplementary Figure 1. Effect of Gwl depletion in oocyte maturation. Prophase I arrested oocytes were injected with HA-TRIM21 mRNA and control or Xenopus Gwl antibodies. Sixteen hours later, oocytes were treated with progesterone and monitored by video. Images were taken at prophase I (GV), GVBD and 16h later. For rescue experiments, oocytes were injected with HA-TRIM21 mRNA Xenopus Gwl antibodies and sixteen hours later injected with the human Gwl protein (hGwl). One hour later, oocytes were treated with Pg and monitored by video. Images were then taken at GVBD and 16h later.

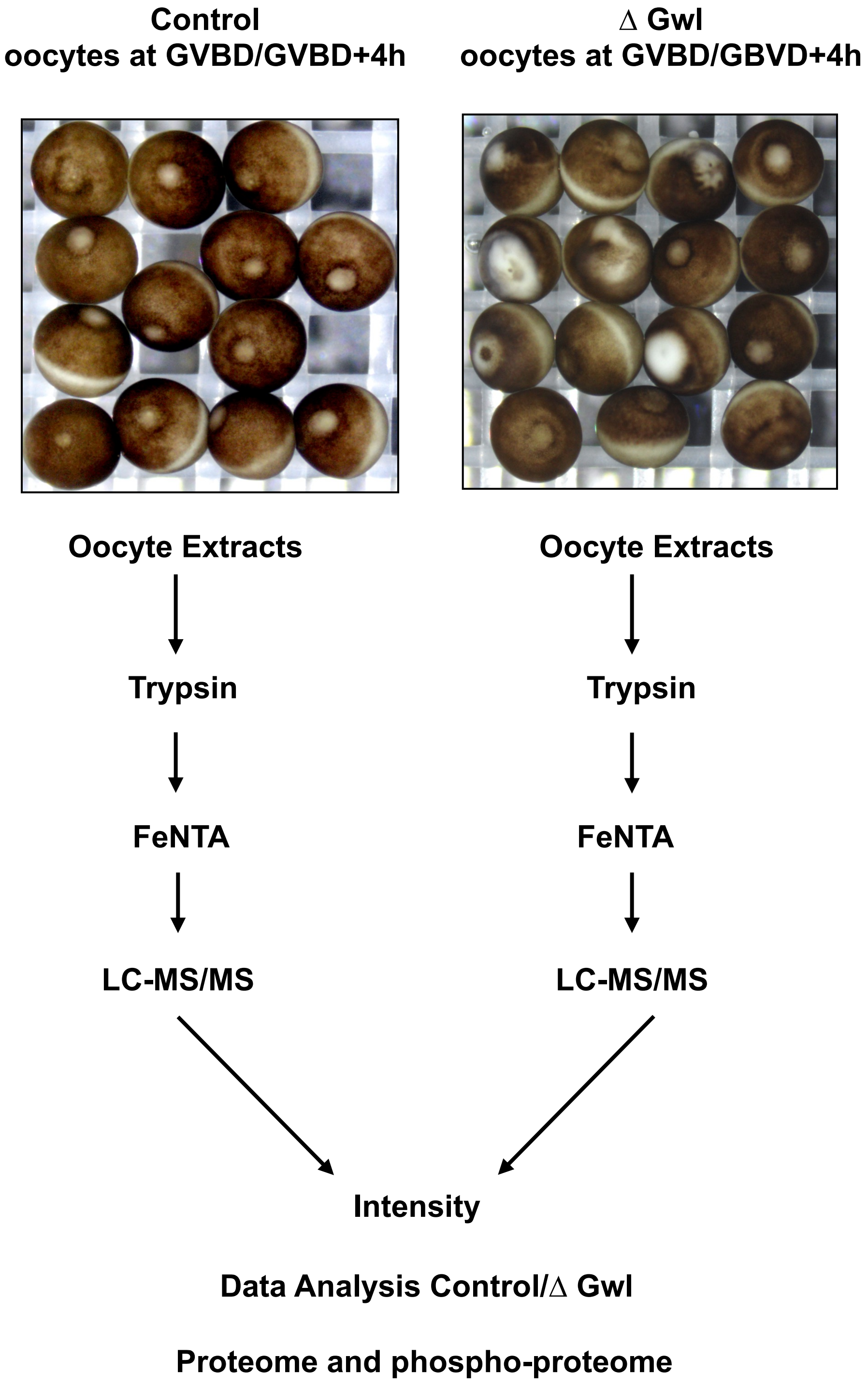

Supplementary Figure 2. Scheme of the protocol used for phosphoproteomic analysis.

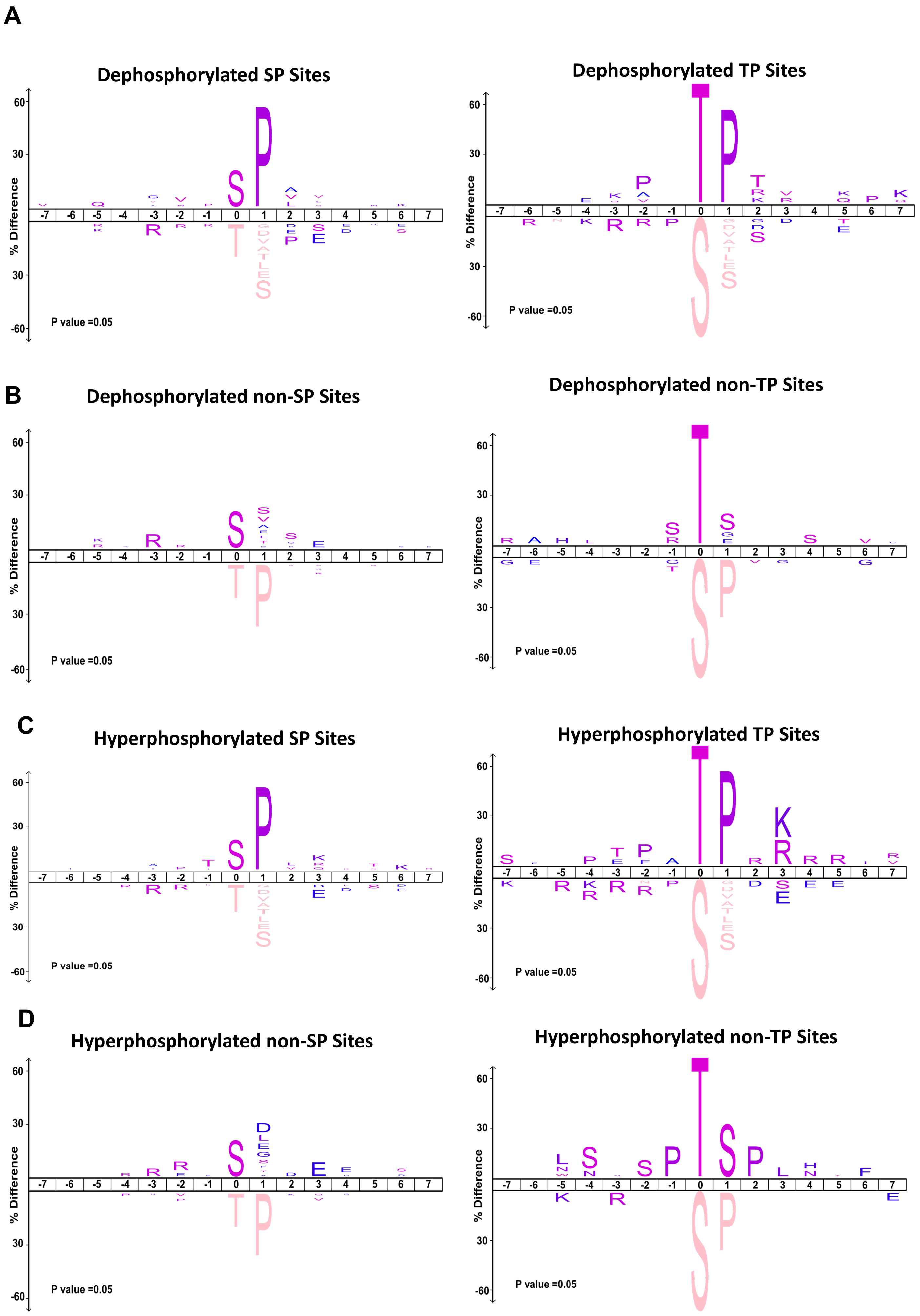
A
Dephosphorylated SP Sites
Dephosphorylated TP Sites
B
Dephosphorylated non-SP Sites
Dephosphorylated non-TP Sites
C
Hyperphosphorylated SP Sites
Hyperphosphorylated TP Sites
D
Hyperphosphorylated non-SP Sites
Hyperphosphorylated non-TP Sites
% Difference
P value =0.05
60
30
30
-60
-7 -6 -5 -4 -3 -2 -1 0 1 2 3 4 5 6 7

Supplementary Figure 3. IceLogo analysis of differentially phosphorylated sites in control and Gwl depleted oocytes. (A) IceLogo analysis with q<0.05 was performed in 15-aminoacid sequences centred on the phosphorylated residue of high confidence SP and TP dephosphorylated sites obtained from GVBD +GVBD+4h injected oocytes (q<0.05; FC<4). (B) As for (A) except that dephosphorylated non-SP and non-TP sites were used. (C) and (D) As for (A) and (B) except that hyperphosphorylated instead of dephosphorylated residues were analysed.

Figure S4

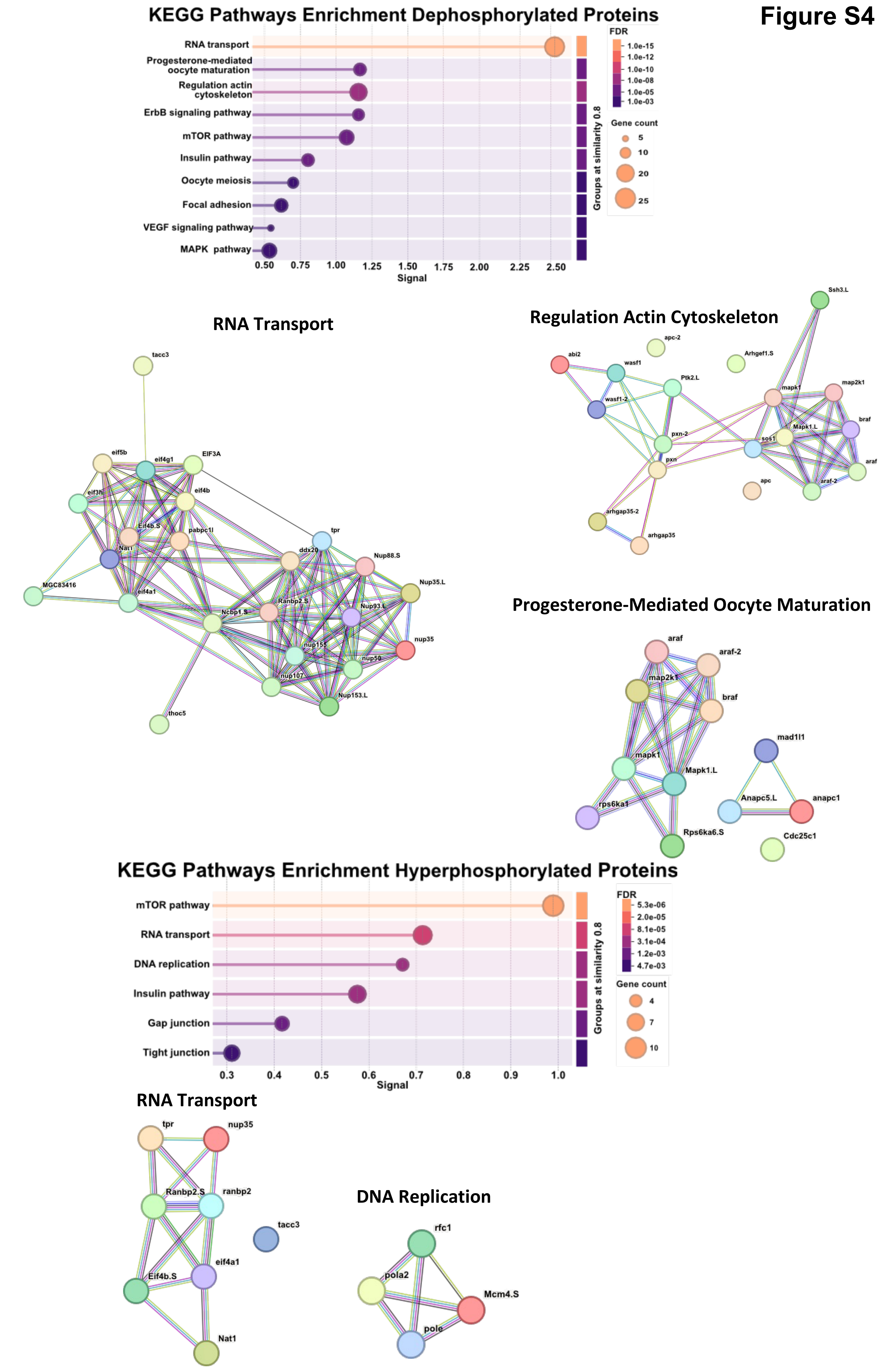

KEGG Pathways Enrichment Dephosphorylated Proteins
RNA transport
Progesterone-mediated oocyte maturation
Regulation actin cytoskeleton
ErbB signaling pathway
mTOR pathway
Insulin pathway
Oocyte meiosis
Focal adhesion
VEGF signaling pathway
MAPK pathway
0.50 0.75 1.00 1.25 1.50 1.75 2.00 2.25 2.50
Signal
Groups at similarity 0.8
FDR
1.0e-15
1.0e-12
1.0e-10
1.0e-08
1.0e-05
1.0e-03
Gene count
5
10
20
25
RNA Transport
tacc3
eif5b
eif4g1
EIF3A
eif3h
eif4b
Eif4b.S
pabpc1l
Nat1
tpr
ddx20
Nup88.S
Nup35.L
MGC83416
eif4a1
Ncbp1.S
Ranbp2.S
Nup93.L
nup35
nup155
nup50
nup107
Nup153.L
thoc5
Regulation Actin Cytoskeleton
Ssh3.L
apc-2
abi2
wasf1
Arhgef1.S
Ptk2.L
mapk1
map2k1
wasf1-2
braf
Mapk1.L
sos1
pxn-2
pxn
araf
araf-2
apc
arhgap35-2
arhgap35
Progesterone-Mediated Oocyte Maturation
araf
araf-2
map2k1
braf
mad1l1
mapk1
Mapk1.L
Anapc5.L
anapc1
rps6ka1
Rps6ka6.S
Cdc25c1
KEGG Pathways Enrichment Hyperphosphorylated Proteins
mTOR pathway
RNA transport
DNA replication
Insulin pathway
Gap junction
Tight junction
0.3 0.4 0.5 0.6 0.7 0.8 0.9 1.0
Signal
Groups at similarity 0.8
FDR
5.3e-06
2.0e-05
8.1e-05
3.1e-04
1.2e-03
4.7e-03
Gene count
4
7
10
RNA Transport
tpr
nup35
Ranbp2.S
ranbp2
tacc3
eif4a1
Eif4b.S
Nat1
DNA Replication
rfc1
pola2
Mcm4.S
pole

Supplementary Figure 4. KEGG pathway enrichment analysis and protein-protein interaction network analysis of the differentially dephosphorylated and hyperphosphorylated proteins were identified using String. KEGG pathway enrichment: bubble size represents gene count; colour indicates-$\log_{10}$(p-value); FDR: False Discovery Rate; Signal: -$\log_{10}$(FDR). Most significant nodes found in String website were represented.

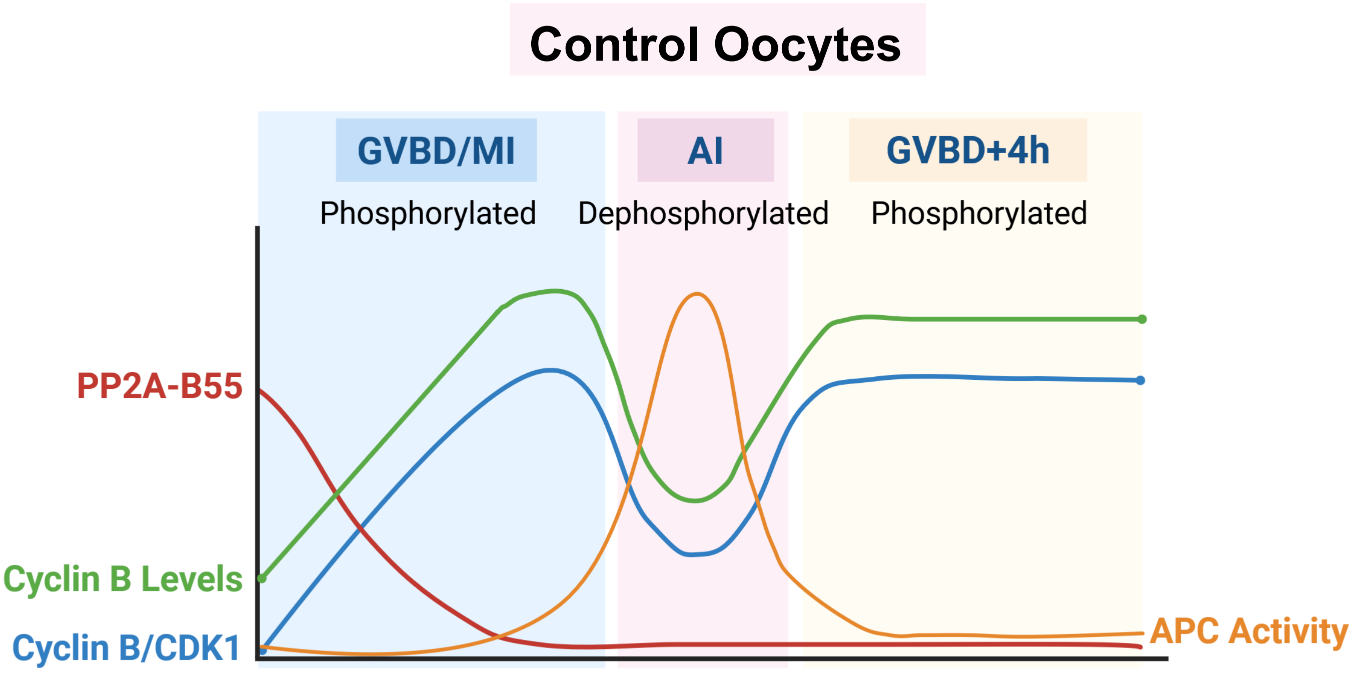
Control Oocytes
GVBD/MI
Phosphorylated
AI
Dephosphorylated
GVBD+4h
Phosphorylated
PP2A-B55
Cyclin B Levels
Cyclin B/CDK1
APC Activity

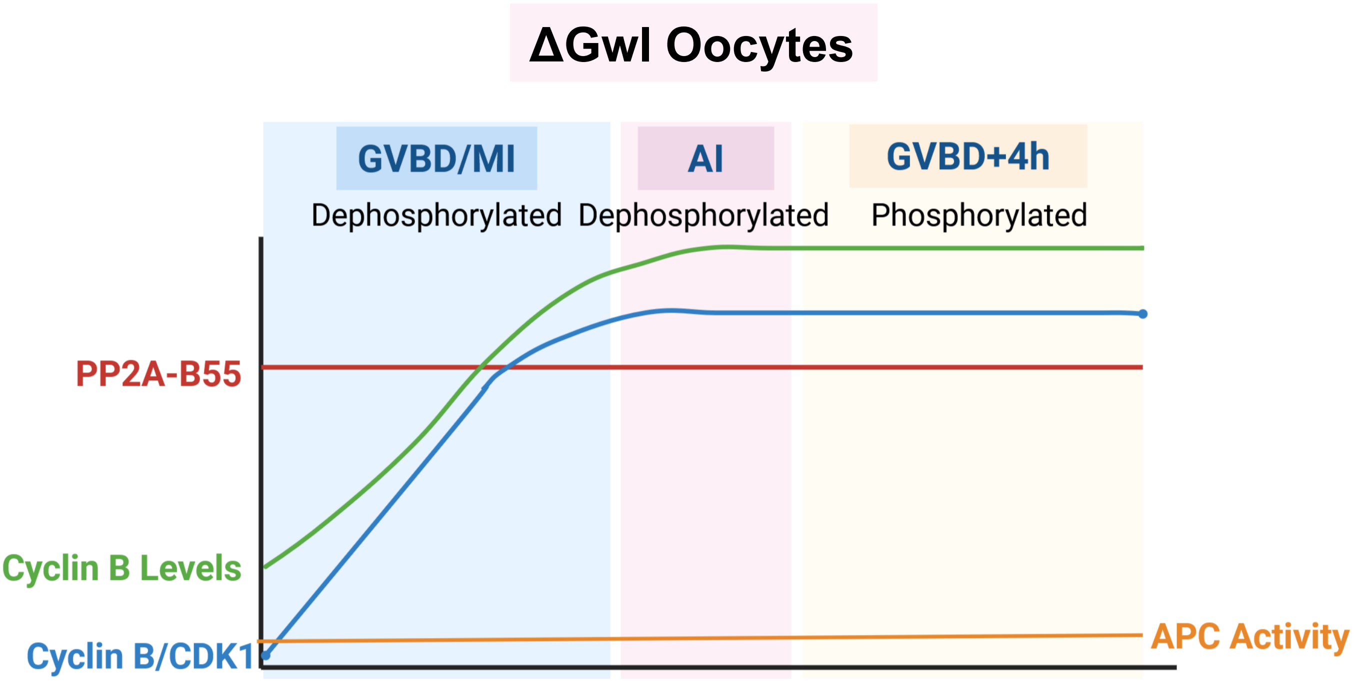
ΔGwl Oocytes
GVBD/MI
Dephosphorylated
AI
Dephosphorylated
GVBD+4h
Phosphorylated
PP2A-B55
Cyclin B Levels
Cyclin B/CDK1
APC Activity

Supplementary Figure 5. Working model explaining the hypophosphorylation of meiotic substrates at GVBD and their hyperphosphorylation at GVBD+4H in Gwl depleted maturing oocytes. Scheme representing the activities of PP2A-B55 and cyclin B/CDK1 in control and Greatwall depleted oocytes as well as the phosphorylation state of meiotic substrates at GVBD/ Metaphase I, Anaphase I and Metaphase II arrested oocytes (GVBD+4H). In Gwl depleted oocytes PP2A-B55 activity remains high throughout meiotic maturation due to the loss of Gwl and dephosphorylated Arpp19 and ENSA. Conversely, cyclin B/CDK1 activity abnormally increases due to the incapacity of the APC/C to degrade cyclin B. However, despite increased cyclin B/ CDK1 activity, this kinase is unable to exceed PP2A-B55 activity at GVBD and substrates remain hypophosphorylated at this stage of meiosis in Gwl devoid oocytes. As oocytes progress in meiosis, cyclin B synthesis proceeds and cyclin B/CDK1 reach abnormal high activity that surpass the activity of the hyperactivated phosphatase promoting substrate hyperphosphorylation at GVBD+4h, a time point in which metaphase II is already established in control oocytes. Created with BioRender.com.

REAGENT or RESOURCE	SOURCE	IDENTIFIER
**Antibodies**		
Mouse monoclonal anti-Xenopus Mos	Santa Cruz	Cat#sc-53373
Rabbit Polyclonal anti-Xenopus Erp1	Vigneron et al. 2009	N/A
Rabbit Polyclonal anti-Xenopus Myt-1	Vigneron et al. 2009	N/A
Rabbit Polyclonal anti-Xenopus Wee-1	Vigneron et al. 2009	N/A
Rabbit Polyclonal phospho-Xenopus pS314-318 APC1	Gift From Hiro Yamano Lab	
Rabbit Polyclonal phospho-Xenopus pS358 APC1	Gift From Hiro Yamano Lab	
Goat Polyclonal Anti Xenopus RSK	Santa Cruz	Cat#sc-1430
Rabbit Polyclonal anti-Human Greatwall	Burgess et al. 2010	N/A
Rabbit Polyclonal anti-Xenopus Greatwall	Vigneron et al. 2009	N/A
Rabbit Polyclonal anti-Xenopus Cdc27	Lorca et al. 2010	N/A
Rabbit Polyclonal Phospho-CdK1 (Tyr-15)	Cell Signalling Technology	Cat# 9111L, RRID:AB_2074655
Rabbit Polyclonal anti-Xenopus Cyclin B2	Abrieu et al. 1997	N/A
Rabbit Polyclonal anti-Xenopus Cdk1	Vigneron et al. 2018	N/A
Rabbit Monoclonal PhosphoThr320 of PP1	Abcam	Cat# 66334
Rabbit Polyclonal anti-Xenopus Plx1	Vigneron et al. 2018	N/A
Rabbit Polyclonal phospho-Human T210 Plk1	Cell Signalling Technology	Cat# 9062S, RRID:AB_11127447
Rabbit Polyclonal anti-Xenopus Cyclin B1	Vigneron et al. 2018	N/A
Rabbit Polyclonal anti-Xenopus Cyclin B3	This study	NA
Rabbit Polyclonal anti-Xenopus Mapk	Abrieu et al. 1996	N/A
Rabbit Polyclonal anti-Xenopus Arpp19	Gharbi et al. 2010	N/A
Rabbit Polyclonal anti-phosphorylated Arpp19 (S67)	Cell Signaling	Cat#5240
HRP conjugated anti-Rabbit secondary antibodies	Cell Signalling Technology	Cat# 7074, RRID:AB_2099233
HRP conjugated anti-Mouse secondary antibodies	BioRad	Cat#1172-1011
Rabbit Polyclonal anti-GST antibodies	Vigneron et al. 2018	N/A
Mouse Monoclonal phospho-T202-Y204 p44/42 Mapk	Cell Signalling Technology	Cat#9106S
Mouse Monoclonal HA-Tag	Cell Signalling Technology	Cat#2367S
Goat anti-Rat IgG (H+L) Cross-Adsorbed Secondary Antibody, Alexa Fluor™ 546	Invitrogen	Cat # A-11081
alpha Tubulin Monoclonal Antibody (YL1/2)	Invitrogen	Cat # MA1-80017
Rabbit anti-GFP	NOVUS	Cat # NB600-308
**Bacterial and Virus Strains**		
BL21DE3 Competent E.Coli	New England Biolabs	Cat#C2527H
DH5α E. Coli	New England Biolabs	Cat#C2987I
**Recombinant Proteins**		

Recombinant (Bacculovirus) GST-Human Greatwall K72M mutant	Vigneron et al. 2011	N/A
**Chemicals, Peptides, and Recombinant Proteins**		
HCG Hormone	Intervet-MSD Sante Animal	Cat#6968493
Pfu ultra II fusion DNA polymerase	Agilent	Cat#600670
Calcium Ionophore	Sigma	Cat#A231987
Dynabeads protein G	Invitrogen	Cat#1009D
ATPγP33	Hartmann Analytic	Cat#FF-301
ATPγS	Sigma	Cat#A1388
Histone H1	Sigma	Cat#14-155
Kemptide	Promega	Cat# V5601
BSA	Sigma	Cat#A7906
Glutathione Sepharose™ hFastFlow	GE Healthcare	Cat#17-5132-02
CNBr-activated sepharose 4B	GE Healthcare	Cat#17-0430-01
TALON Superflow Metal Affinity Resin	Takara	Cat#635506
Amylose Resin High Flow	Biolabs	Cat#E80225
PVDF Transfert Membrane	Millipore	Cat#88518
Sulfo-MBS	Thermo Scientific	Cat#22312
Epoximicin	Euromedex	Cat#F1400-1-UBP
SYTOX™ Green - Solution	Invitrogen	Catalog #S7020
Reticulocyte lysate system	Promega	Cat# L4960
mMESSAGEmMACHINE SP6	INVITROGEN	Cat#AM1340
**Experimental Models: Organisms/Strains**		
Xenopus Laevis	TEFOR Paris-Saclay	https://tefor.net/
**Oligonucleotides**		
Site-Directed Mutagenesis for Cyclin B3 R51A: forward and reverse primers:<br>5’ CAG-GGA-GGT-GCA-AAG-AAG-GCA-GCA-GCA TTT-GGG-GAC-ATT-AC 3’<br>5’ GT-AAT-GTC-CCC-AAA-TGC-TGC-TGC-CTT-CTT TGC-ACC-TCC-CTG 3’	Eurogentec	This study
Amplification of Xenopus Cyclin B3 from Xenopus ovary cDNA; forward and reverse primers:<br>5' ATGATGCCTTCTCTTCGTCCATC 3'<br>5' TATTAGCTCTGAAGGGCCTCTG 3'	Eurogentec	This study
**Sequences**		
5’mos GFP 3’ mos<br>ctgcagtgagattgaggggaaggcgcacgccggctatttatacatgtgagcg cgtcggtccctcccccagtagcgtcgcattttaccggcgcatccgtttcagcga gtcacacagcaATGGTGAGCAAGGGCGAGGAGCTGT TCACCGGGGTGGTGCCCATCCTGGTCGAGCTGG ACGGCGACGTAAACGGCCACAAGTTCAGCGTGT CCGGCGAGGGCGAGGGCGATGCCACCTACGGC AAGCTGACCCTGAAGTTCATCTGCACCACCGGC AAGCTGCCCGTGCCCTGGCCCACCCTCGTGACC ACCCTGACCTACGGCGTGCAGTGCTTCAGCCGC TACCCCGACCACATGAAGCAGCACGACTTCTTC AAGTCCGCCATGCCCGAAGGCTACGTCCAGGAG CGCACCATCTTCTTCAAGGACGACGGCAACTAC	This Study	Genecust

AAGACCCGCGCCGAGGTGAAGTTCGAGGGCGA<br>CACCCTGGTGAACCGCATCGAGCTGAAGGGCAT<br>CGACTTCAAGGAGGACGGCAACATCCTGGGGC<br>ACAAGCTGGAGTACAACTACAACAGCCACAAC<br>GTCTATATCATGGCCGACAAGCAGAAGAACGG<br>CATCAAGGTGAACTTCAAGATCCGCCACAACAT<br>CGAGGACGGCAGCGTGCAGCTCGCCGACCACT<br>ACCAGCAGAACACCCCCATCGGCGACGGCCCC<br>GTGCTGCTGCCCGACAACCACTACCTGAGCACC<br>CAGTCCGCCCTGAGCAAAGACCCCAACGAGAA<br>GCGCGATCACATGGTCCTGCTGGAGTTCGTGAC<br>CGCCGCCGGGATCACTCTCGGCATGGACGAGCT<br>GTACAAGtgacgtccagaacagggagccaatcagcactgtgccacag<br>gacatataaagccaggccagcgatctcatcattaatgtgttgggggggacacat<br>gcccagggcagcgtcacaggcttccactccactcaatcccagctcagtcgtgc<br>actagtgagggagcatcactccttgtcctatctgcttgcccagatgatctgaaat<br>agtggccaggatctcttgcatcttccttcttatcaattgaaaactttaaagatgaga<br>tgtcattggtcgctttttcctgtgcctgagacatttggtctgtgatgtagacaatga<br>ctataatgtcagcggggagagctttgcttttgcacggccaaactttacattgcatt<br>agccatagagggtttttgtgtgtgacttgtggcactctgtcacatatatacgttttg<br>cacttttcattgtctgctttctttttttaatgttgggctccatttagtctactggtttggat<br>ggggttaattcaatttccatggggcaattcataccaagtgcactatcctatgttcc<br>gttattgtattaccccagctaatctacaagacgtacaaagcatatggcagaatta<br>cactccaatcagaagtgtgaatgggcctttttcagtgttttatatgtatgtgttgtttt<br>atgtgtgtgtgtgtgctcttctctcctgcacaatgtgttatgttgatccatgtaggaa<br>ataagatcacaaagcagtcattgcatatagtcctgtacttcagttttcgtatccccc<br>tcttgttcccttagttacttgtaagttcaagtgcagtcataggtggtagtagtggatt<br>gtggcatttgtcatttagagggagcaaagctttattattcaaggctatacgcagg<br>gatcagcagcctttggccattgcagaaggaaacacccagaaggcaacagata<br>agttccacagcaacccggagactgcttgttccatgtataagacttttatatttttctc<br>gggtaacaaatgcacagtctcttgctaatctagtacagtatctccatgtgcattaa<br>tttatattaaaactccttcgtccatagtgtcattctctgatcggtagatcggattaat<br>gtccctacatattatagtagaagtacagtccacaaaatagatgttgtgcccagta<br>atttaactattgaacccactgtgtccaccagtctcgtttgtagccgtctgttcccttg<br>tgcaaaaaaataaatctttatggtagaaatacataaataagtttaaattaagccaa<br>cactgtgaaatgtagaacatcacaaatgccctccccccttccactttagaatgtaat<br>gtctttactctttaaaacagtgatgcagatatatactgcaaacatctctggcagtca<br>cacggttaaacatcagctgtgcaactgcattttcctgctgtctaaattaattccaac<br>acctggggatttcataaattgcttctgttgagggaactgtcttgggacagcttgtg<br>tcctctgcacattggccccagcagaagtctctctggagcagggagtgcaacct<br>gaatacagcaatgtccctggggaatcactagtagccaggagttcatatgtgact<br>tcagtggttgccaaacacacagggctatattctctaaaccagggtgtccagcct<br>gtggctgtacagttttcgaatggactttagcagctggcatgttcagtcttcctttga<br>taggatgccgggagttgtgggtttgtgtgaatgactgcctttactgttgctttatag<br>cactgaaaatacaagcaaggatatgaaaaaaagatttcattttaaaaatgtgttg<br>cattgctgtttaagtggtaatattccaaagtgaatatatagttttataataaagaaat<br>tgatttgtct		
**Recombinant DNA**		
pGEX4T2 Cyclin B3 Xenopus	This Study	N/A
pMalP2 Cyclin B3 Xenopus	This Study	N/A
pCS2 Cyclin B3 Xenopus	This Study	N/A
HA-pCS2 TRIM21 Human	This Study	N/A
HA-pCS2 Wee1 Xenopus	This Study	N/A

HA-pCS2 Myt1 Xenopus	This Study	N/A
HA-pCS2 GFP Pseudomonas aeruginosa	This Study	N/A
**Software and Algorithms**		
Adobe Photoshop	Microsoft	Version 12.0x64
Excel	Microsoft	Version 14.7.7
PowerPoint	Microsoft	Version 14.7.7
ImageJ	NIH	1.50i
GraphPad Prism	Prism 10 for macOS	Version 10.5.0

References :

1. Abrieu et al. MAPK inactivation is required for the G2 to M-phase transition of the first mitotic cell cycle. The EMBO Journal (1997) 16, 6407-6413.
2. Abrieu et al. The Polo-like kinase Plx1 is a component of the MPF amplification loop at the G2/M-Phase transition of the cell cycle in Xenopus eggs. Journal of Cell Science (1998) 111, 1751-1757.
3. Burgess el al. Loss of human Greatwall results in G2 arrest and multiple mitotic defects due to deregulation of the cyclin B-Cdc2/PP2A balance. PNAS (2010) 107, 12564–12569.
4. Gharbi-Ayachi et al. The substrate of Greatwall kinase, Arpp19, controls mitosis by inhibiting protein phosphatase 2A. Science (2010) DOI: 10.1126/science.1197048.
5. Lorca et al. Constant regulation of both the MPF amplification loop and the Greatwall-PP2A pathway is required for metaphase II arrest and correct entry into the first embryonic cell cycle. doi:10.1242/jcs.064527
6. Vigneron et al. Characterization of the Mechanisms Controlling Greatwall Activity. MOLECULAR AND CELLULAR BIOLOGY (2011), doi:10.1128/MCB.00753-10.
7. Vigneron et al. Greatwall maintains mitosis through regulation of PP2A. The EMBO Journal (2009) 28, 2786–2793
8. Vigneron et al. Cyclin A-cdk1-Dependent Phosphorylation of Bora is the Triggering Factor Promoting Mitotic Entry Developmental Cell (2018) 45, 637-650.
