# Supplementary material for: Greatwall depletion from Xenopus oocytes reveals a key role of the cyclin B/CDK1-PP2A-B55 balance in the coordination of meiotic events
Source: Nat Commun. 2026 May 13;17:6361. doi: 10.1038/s41467-026-73011-5 (PMC13376403; doi:10.1038/s41467-026-73011-5)
Supplement: Supplementary file 3 — Transparent Peer Review file [file 41467_2026_73011_MOESM3_ESM.pdf]

nature portfolio

Peer Review File

Greatwall depletion from Xenopus oocytes reveals a key role of the cyclin B/CDK1-PP2A-B55 balance in the coordination of meiotic events

Corresponding Author: Dr Thierry Lorca

This file contains all reviewer reports in order by version, followed by all author rebuttals in order by version.

Version 0:

Reviewer comments:

Reviewer #1

(Remarks to the Author)
General assessment

The authors utilise the TRIM-Away technique to deplete Greatwall (Gwl) kinase in prophase I arrested Xenopus oocytes and assess the impact of Gwl depletion on the meiotic phosphoproteome and functionality of signalling circuits required for efficient metaphase II block.

In the first part of the manuscript, the authors demonstrate that depletion of Gwl does not prevent the activation of CDK1-CycB, allowing progression through meiosis. This observation is interesting, as depletion of Gwl blocks G2/M transition in G2 Xenopus egg extracts. The authors argue this discrepancy could be a consequence of different expression patterns of WEE1 kinase, which is absent in prophase I oocyte, but expressed in G2.

In the second part of the manuscript, the authors characterise the impact of Gwl loss on the meiotic phosphoproteome.

Phosphoproteomic analysis leads to the third part of the manuscript, where the authors functionally integrate Gwl activity into a meiotic signalling network. The authors demonstrate that Gwl is required for the robust phosphorylation and activation of APC, which drives the degradation of cyclins B1, B2 and B3. Additionally, the authors show that Gwl plays a role in the stabilisation of c-Mos required for the activation of the MEK/MAPK1/Rsk pathway. Although the authors debate the mechanism of Gwl-mediated c-Mos regulation, this is not experimentally addressed. Combined dysregulation of CDK1-CycB3 and Rsk1/2 prevents the sufficient accumulation of the APC inhibitor Erp1 required for the metaphase II block.

Although the requirement of Greatwall kinase for meiotic APC activation has been discussed elsewhere (PMID: 25246615), the authors enhance our understanding of Greatwall function and the implications of PP2A-B55 dysregulation on meiotic progression. The authors generated a comprehensive phosphoproteomic dataset, which will represent a valuable resource. The observation that Gwl plays a role in c-Mos stabilisation is novel.

Methodology

The study employs standard methodologies, including imaging, Western Blot-based assessment of protein and phospho-protein quantities, phosphoproteomics, and in vitro kinase activity assays. Utilised methods are described in sufficient detail.

Data and code availability

I was unable to find links to the data (especially the phosphoproteomics) and the code used to analyse them. Thus, I could not assess the credibility of the presented analyses.

Suggestions to improve the manuscripts

The manuscript could be improved by expanding the phosphoproteomic analysis, as indicated in the comments below. I

also recommend the implementation of additional fluorescence microscopy techniques to ascertain the impact of Gwl depletion on meiotic chromosomal organisation. A significant portion of the manuscript is based on Western Blot analysis. The authors show representative blots. I understand some Western Blots can be difficult or impossible to quantify. The authors, however, should find a way (quantification or other) to demonstrate the reproducibility of the data. This is critical to allow the reader to assess the robustness of the presented analyses and conclusions.

Additional comments and corrections

1. In Supplementary Fig. 1C, it is not clear what the label “GVBD-ON” refers to. Based on the accompanying legend, I assume this is 16 hours after GVBD. The label should be changed to make this clearer.

2. In line 128, the authors state that overexpression of Myt1 in ΔGwl oocytes allows maturation. It should also be said that this maturation is significantly delayed, as indicated by Fig. 1D.

3. In Fig. 1E, in both control and ΔGwl conditions, PI and GV+2h time points show high levels of CDK1Y15-P. The phosphorylation levels of CDK1Y15-P appear to be decreased when Wee1 and Myt1 are overexpressed. This seems to be counterintuitive as Wee1 and Myt1 phosphorylate CDK1Y15. The authors should clarify this discrepancy.

4. In line 155, “(49,6 % vs 51,7 respectively)” should be corrected for better readability and consistency. (49.6 % vs 51.7%, respectively) would probably be better.

5. In lines 158-160, the authors state that depletion of Gwl had only a minor impact on global protein levels. This conclusion should be supported by evidence. Assuming the authors have such data, I suggest including a graph indicating the changes in global protein levels following Gwl depletion.

6. In lines 160-161, the authors should show changes in cyclin B levels.

7. In lines 172-175, the authors state that depletion of Gwl disproportionally affected phosphorylation of serine and threonine residues. A graph should support this conclusion.

8. In Fig. 3C, the authors combine differentially-phosphorylated threonine and serine residues to show distinct tendencies towards dephosphorylation and hyperphosphorylation at GVBD and GVBD+4h. In lines 177-179, the authors argue that these differential tendencies are driven by preferential dephosphorylation of threonine residues by PP2A-B55. This should be supported by a new graph derived from the presented phosphoproteomic data. Alternatively, the authors could modify Fig. 3C to display serine and threonine residues separately. Would such data presentation be consistent with the reasoning?

9. In lines 179-181, the authors state that the tendencies for hyperphosphorylation and dephosphorylation differed when only proline-neighbouring phosphosites were considered. A graph should support this.

10. In Fig. 3D, the authors present a KEGG analysis of dephosphorylated and hyperphosphorylated hits. The two given graphs are duplicated, only showing KEGG enrichment of dephosphorylated peptides. This must be corrected.

11. The authors should consider including a figure listing a subset of differentially phosphorylated peptides of potential interest. These could be, for example, proteins involved in the regulation of the meiotic cell cycle, the mitotic cell cycle, chromosomal organisation, etc.

12. In lines 206-209, amino acid residues should be referred to consistently, either by a single-letter or three-letter code. This should be consistent with the rest of the text.

13. In Fig. 6A, I recommend using a monospaced font, such as Courier, which enhances the readability of the protein alignment.

14. In Fig. 4B (line 786) and in Fig. 6D (line 830) legends, “pick intensity” should be corrected to “peak intensity”.

15. In Fig. 5B and Fig. 7B, to demonstrate the reproducibility of these assays, the authors should present the results of at least three independent experiments, along with their means, standard deviations and appropriate statistical analysis.

16. In Supplementary Fig. S1, control oocytes are predominantly brown, and ΔGwl oocytes are white. Could the authors comment on what this indicates? For someone unfamiliar with this experimental system, this may be unclear.

17. Indication of decimal points should be consistent across the manuscript. Authors sometimes use a comma and sometimes a period.

18. Authors sometimes, but not always, separate thousands by a comma. This should be consistent across the manuscript.

Reviewer #2

(Remarks to the Author)
This paper investigates the role of the kinase Greatwall (Gwl) in the meiotic maturation of Xenopus oocytes, a well characterized model to study the meiotic divisions. Using the Trim-Away approach to induce the degradation of the Gwl protein that is expressed in the oocytes, it is shown that Gwl-deficient oocytes enter meiotic I normally upon progesterone stimulation but then are unable to complete the meiotic divisions. Two major biochemical changes are found upon Gwl depletion. One is the failed accumulation of the kinase Mos, which results in impaired activation of the MAPK-Rsk1/2 pathway preventing metaphase II arrest. In addition, several subunits of the APC/C complex are not phosphorylated and APC/C remains inactive during the meiosis I-meiosis II transition, impairing cyclin degradation and the partial CDK1 inactivation that is required at this stage. The combined lack of Rsk1/2 activation and cyclin B3 degradation during meiosis I prevents the accumulation of the APC/C inhibitor Erp1/Emi2 and oocytes fail to arrest at meiosis II. Taken together, the results support an important role for Gwl in the coordination of the meiotic divisions, acting through dual regulation of the Mos/MAPK/Rsk1/2 and APC/C pathways.

This is an interesting study that contains high-quality biochemical data, which altogether support the idea that Gwl plays a key role in the regulation of the meiotic divisions. The Trim-Away system provides a neat approach to address the role of Gwl as well as to perform rescue experiments using purified proteins or combined downregulation of two proteins to explore the underlying mechanisms. In my opinion, the manuscript provides an interesting contribution to the field but some conclusions should be better supported. Below are a few points that should be addressed to make the story more convincing.

1. The authors and others have previously shown that a key function of Gwl is to negatively regulate PP2A-B55 activity. However, although mentioned several times, there is no evidence in the manuscript that the effects observed upon Gwl depletion in oocytes are due to the hyperactivation of PP2A-B55. A rescue experiment combining Gwl depletion with PP2A-B55 inhibition or inactivation would support this conclusion.

2. Lack of Mos accumulation seems to be an important effect of Gwl depletion in progesterone-treated oocytes together with the regulation of APC/C activity. But while APC/C regulation is study in more detail no information is provided on the regulation of Mos by Gwl. At the very least, it should be investigated whether Mos accumulation is controlled by Gwl at the translation or protein stability levels.

3. Fig S1C shows that Gwl-depleted oocytes undergo meiosis I entry (GVBD) but then clearly degenerate, either because they are unable to transition from meiosis I to meiosis II, or to arrest in meiosis II. However, there is information on what happens to the oocytes when the biochemical effects observed upon Gwl-depletion are rescued by different means (addition of Gwl and Mos or depletion of Cdc27 and cyclin B3). This should be illustrated in all cases as for Gwl-depletion in S1C.

4. Fig S1A and S1B can be removed as exactly the same information is provided in Fig 1B. Likewise, Fig 4D can be removed as Fig 4E contains the same information plus a couple of additional blots.

5. Fig. 3 is of limited interest and could be moved to Supplementary. Instead, it would be nice to include more information on what was actually learned from the phospho-proteomics experiment performed. If about 40% of the differential phosphorylations detected upon Gwl-depletion do not correspond to Cdk/cyclin consensus motifs sites, what are the predicted kinases controlling the observed dephosphorylations? How are the hyperphosphorylations occurring upon Gwl depletion?

6. Fig. 3D, both panels indicate Dephosphorylated proteins but maybe one of them should indicate KEGG with Phosphorylated proteins?

7. Fig. 7D, the restored accumulation of Erp1 upon combined depletion of Cdc27 and cyclin B3 is not very clear in the blot presented.

8. The model of Fig. S2 could be elaborated a bit more and included as a figure in the main manuscript.

9. The choice of references is a bit odd and should be revised. For example, in page 6: “During meiotic progression, c-Mos is translated at GVBD promoting the activation of the MAP Kinase Kinase 1, MEK1, and the final phosphorylation of MAPK1 (ref. 17)”. In fact, ref. 17 has nothing to do with c-Mos translation at GVBD nor with MAPK1 activation in oocytes.

Version 1:

Reviewer comments:

Reviewer #1

(Remarks to the Author)
I have read the author's response letter and the updated version of the manuscript. The authors have done a good job of addressing the comments and revising the manuscript, including the introduction of a completely new section on meiotic chromosomal defects associated with Greatwall depletion. I believe the resubmitted work is of adequate quality and will serve as a valuable resource for researchers interested in meiotic division and Greatwall/PP2A-B55 signalling. I recommend this work for publication in the journal Nature Communications.

Reviewer #2

(Remarks to the Author)
The new version of the manuscript addresses all my concerns, mostly by providing additional data that support the authors' conclusions. I therefore recommend publication of the revised manuscript.

As a minor point, the new text (marked in blue) contains a few typos and should be edited.

Open Access This Peer Review File is licensed under a Creative Commons Attribution 4.0 International License, which permits use, sharing, adaptation, distribution and reproduction in any medium or format, as long as you give appropriate credit to the original author(s) and the source, provide a link to the Creative Commons license, and indicate if changes were made.
In cases where reviewers are anonymous, credit should be given to 'Anonymous Referee' and the source.
The images or other third party material in this Peer Review File are included in the article's Creative Commons license, unless indicated otherwise in a credit line to the material. If material is not included in the article's Creative Commons license and your intended use is not permitted by statutory regulation or exceeds the permitted use, you will need to obtain permission directly from the copyright holder.
To view a copy of this license, visit https://creativecommons.org/licenses/by/4.0/

Dear Editor,

Enclosed you will find the revised version of our manuscript entitled "Greatwall depletion from Xenopus oocytes reveals a key role of cyclin B/CDK1-PP2A-B55 balance I the coordination of meiotic events" by Roque et al. (NCOMMS-25-58616) that we would like to resubmit to Nature Communications in which all the concerns of the referees have been addressed.

We thank the referees for their positive comments and valuable advices, which helped us to improve the quality and the clarity of our manuscript. Source Data are provided with this paper.

REFERRE #1

Suggestions to improve the manuscript

1- "The manuscript could be improved by expanding the phosphoproteomic analysis, as indicated in the comments below".

The phosphoproteomic analysis has been improved by considering the comments of the two referees (see below).

2- "I also recommend the implementation of additional fluorescence microscopy techniques to ascertain the impact of Gwl depletion on meiotic chromosomal organisation".

Immunofluorescence experiments to investigate the effect of Gwl depletion in spindle and chromosome structure in maturing oocytes have been implemented and included in Figure 8. These data demonstrate that the loss of Gwl impairs correct chromosome condensation and prevents spindle formation and spindle migration to the cortex. This phenotype correlate with the presence in our phosphoproteome of dephosphorylated proteins involved in microtubule polymerization and actin cytoskeleton regulators as well as proteins involved in chromosome condensation.

3- "A significant portion of the manuscript is based on Western Blot analysis. The authors show representative blots. I understand some Western Blots can be difficult or impossible to quantify. The authors, however, should find a way (quantification or other) to demonstrate the reproducibility of the data. This is critical

to allow the reader to assess the robustness of the presented analyses and conclusions”.

Levels of proteins showing substantial quantitative differences in Figure 4D, Figure 5C, Figure 5D, Figure 7A and Figure 7D have been quantified in three different experiments and represented as mean+/- standard deviation.

Additional comments and corrections

1-Point 1:

“In Supplementary Fig. 1C, it is not clear what the label “GVBD-ON” refers to. Based on the accompanying legend, I assume this is 16 hours after GVBD. The label should be changed to make this clearer”

“GVBD-ON” referred to GVBD-Over Night. We modified this label to “GVBD+16h” to ensure consistency with the rest of the labels and to improve clarity.

Point 2:

" In line 128, the authors state that overexpression of Myt1 in ΔGwl oocytes allows maturation. It should also be said that this maturation is significantly delayed, as indicated by Fig. 1D."

This statement was already present in lanes 126-127 of the previous version of the manuscript and has been highlighted in bold in lanes 132-133 of the revised version.

Point 3:

" In Fig. 1E, in both control and ΔGwl conditions, PI and GV+2h time points show high levels of CDK1Y15-P. The phosphorylation levels of CDK1Y15-P appear to be decreased when Wee1 and Myt1 are overexpressed. This seems to be counterintuitive as Wee1 and Myt1 phosphorylate CDK1Y15. The authors should clarify this discrepancy. "

The CDK1Y15-P western blot for these samples has been repeated and the new image replaces the original one.

Point 4:

" In line 155, “(49,6 % vs 51,7 respectively)” should be corrected for better readability and consistency. (49.6 % vs 51.7%, respectively) would probably be better."

Commas have been corrected to periods.

Point 5:

" In lines 158-160, the authors state that depletion of Gwl had only a minor impact on global protein levels. This conclusion should be supported by evidence. Assuming the authors have such data, I suggest including a graph indicating the changes in global protein levels following Gwl depletion. "

A volcano plot showing the differences in global protein levels of control vs Gwl depleted oocytes has been added in Figure 2D. The position of cyclin B in this volcano plot has been indicated.

Point 6:

" In lines 160-161, the authors should show changes in cyclin B levels. "

As indicated above, the position of cyclin B in the volcano plot of global protein level differences in control vs Gwl depleted oocytes has been shown in Figure 2D.

Point 7:

" In lines 172-175, the authors state that depletion of Gwl disproportionally affected phosphorylation of serine and threonine residues. A graph should support this conclusion. "

Figure 3C in the original version pSP vs. total pS was plotted alongside with pTP vs. total pT in a single panel, which, as pointed out by the referee, was confusing and risked misinterpretation. We have now split it into two distinct panels in the revised manuscript.

Point 8:

" In Fig. 3C, the authors combine differentially-phosphorylated threonine and serine residues to show distinct tendencies towards dephosphorylation and hyperphosphorylation at GVBD and GVBD+4h. In lines 177-179, the authors argue that these differential tendencies are driven by preferential dephosphorylation of threonine residues by PP2A-B55.

This should be supported by a new graph derived from the presented phosphoproteomic data. Alternatively, the authors could modify Fig. 3C to display serine and threonine residues separately. Would such data presentation be consistent with the reasoning? "

As reported above, serine and threonine residues were represented in two different graphs in the revised version.

Point 9:

" In lines 179-181, the authors state that the tendencies for hyperphosphorylation and dephosphorylation differed when only proline-neighbouring phosphosites were considered. A graph should support this."

Figure 3C and 3D of the revised version of the manuscript clearly demonstrate that the percentages of dephosphorylation/hyperphosphorylation of pSP vs pS and pTP vs pT at GVBD and GVBD+4h are inversed, with a higher percentage of dephosphorylation at GVBD and a of hyperphosphorylation at GVBD+4h.

Point 10:

" In Fig. 3D, the authors present a KEGG analysis of dephosphorylated and hyperphosphorylated hits. The two given graphs are duplicated, only showing KEGG enrichment of dephosphorylated peptides. This must be corrected."

We thank the referee for pointing out this error. Figure S4 in the revised manuscript presents the two KEGG analyses (dephosphorylated and hyperphosphorylated hits) alongside STRING interactome networks for proteins in the most representative functional families.

Point 11:

" The authors should consider including a figure listing a subset of differentially phosphorylated peptides of potential interest. These could be, for example, proteins involved in the regulation of the meiotic cell cycle, the mitotic cell cycle, chromosomal organisation, etc. "

As reported in the previous point, a list of the main phosphorylated/dephosphorylated proteins of the most representative functional families have been represented as String networks in Figure S4.

Point 12:

" In lines 206-209, amino acid residues should be referred to consistently, either by a single-letter or three-letter code. This should be consistent with the rest of the text."

Amino acid residues have been modified.

Point 13:

"In Fig. 6A, I recommend using a monospaced font, such as Courier, which enhances the readability of the protein alignment".

Figure 6A has been modified.

Point 14:

"In Fig. 4B (line 786) and in Fig. 6D (line 830) legends, "pick intensity" should be corrected to "peak intensity".

"Pick" has been modified to "Peak"

Point 15:

"In Fig. 5B and Fig. 7B, to demonstrate the reproducibility of these assays, the authors should present the results of at least three independent experiments, along with their means, standard deviations and appropriate statistical analysis".

Kinase activity values in Figure 5B and 7B have been represented as the mean +/- standard deviation of three different experiments. Statistical differences have also been shown.

Point 16:

"In Supplementary Fig. S1, control oocytes are predominantly brown, and ΔGwl oocytes are white. Could the authors comment on what this indicates? For someone unfamiliar with this experimental system, this may be unclear".

Xenopus oocyte color evolves from brown to white when they degenerate.

Point 16:

"Indication of decimal points should be consistent across the manuscript. Authors sometimes use a comma and sometimes a period".

Decimal points have been corrected.

Point 18:

"Authors sometimes, but not always, separate thousands by a comma. This should be consistent across the manuscript.

Thousands have been consistently separated by a point throughout the manuscript.

REFERRE #2

Point 1:

" The authors and others have previously shown that a key function of Gwl is to negatively regulate PP2A-B55 activity. However, although mentioned several times, there is no evidence in the manuscript that the effects observed upon Gwl depletion in oocytes are due to the hyperactivation of PP2A-B55. A rescue experiment combining Gwl depletion with PP2A-B55 inhibition or inactivation would support this conclusion."

A rescue experiment of the main phenotype observed upon Gwl loss has been performed by microinjecting Gwl depleted oocytes with thio-phosphorylated Arpp19 in the Gwl site S71. Thio-phosphorylation prevents or drastically delays dephosphorylation. The presence of stably S71-phosphorylated Arpp19 in Gwl depleted oocytes inhibits hyperactivated PP2A-B55 restoring its normal activity. As shown in Figure 5E, restoration of PP2A-B55 activity to normal levels by thio-S71-phospho-Arpp19 fully rescued c-Mos levels, MAPK activity and the degradation of cyclin B2 and cyclin B3 supporting that the observed phenotypes are induced by the hyperactivation of PP2A-B55 resulting from Gwl depletion.

Point 2:

"Lack of Mos accumulation seems to be an important effect of Gwl depletion in progesterone-treated oocytes together with the regulation of APC/C activity. But while APC/C regulation is study in more detail no information is provided on the regulation of Mos by Gwl. At the very

least, it should be investigated whether Mos accumulation is controlled by Gwl at the translation or protein stability levels."

New data about the mechanisms preventing c-Mos accumulation in Gwl depleted maturing oocytes have been added in Figure 4E and 4F. Our data demonstrate that c-Mos translation is not affected whereas the loss of Gwl results in the proteasome-dependent degradation of the former protein indicating that Gwl is essential for the stabilization of c-Mos.

Point 3:

" Fig S1C shows that Gwl-depleted oocytes undergo meiosis I entry (GVBD) but then clearly degenerate, either because they are unable to transition from meiosis I to meiosis II, or to arrest in meiosis II. However, there is information on what happens to the oocytes when the biochemical effects observed upon Gwl-depletion are rescued by different means (addition of Gwl and Mos or depletion of Cdc27 and cyclin B3). This should be illustrated in all cases as for Gwl-depletion in S1C."

Picture of oocytes in the different rescue experiments have been added. This includes, Figure S1 (rescue of Gwl devoid oocytes with human Gwl protein), Figure 5A (rescue of ΔGwl oocytes with c-Mos protein), Figure 5D (rescue of ΔGwl+ΔCyB3 oocytes with c-Mos protein) and Figure 7D (triple ΔGwl+ΔCdc27+ΔCyB3 oocytes).

Point 4:

" Fig S1A and S1B can be removed as exactly the same information is provided in Fig 1B. Likewise, Fig 4D can be removed as Fig 4E contains the same information plus a couple of additional blots."

These western blots have been eliminated.

Point 5:

" Fig. 3 is of limited interest and could be moved to Supplementary. Instead, it would be nice to include more information on what was actually learned from the phospho-proteomics experiment performed. If about 40% of the differential phosphorylations detected upon Gwl-depletion do not correspond to Cdk/cyclin consensus motifs sites, what are the predicted kinases controlling the observed

dephosphorylations? How are the hyperphosphorylations occurring upon Gwl depletion?."

Figure 3 has been modified. KEGG analyses have been moved to Supplementary Figure S4 and expanded by the addition of String interactome networks of phosphorylated/dephosphorylated hits in the most enriched functional families. Moreover, IceLogo enriched sequence analyses of phosphorylated/dephosphorylated pSP/pTP and non-pSP/pTP peptides have been implemented to investigate the putative phosphatases/kinases that could be involved in these phosphorylations/dephosphorylations. These new data are depicted in Supplementary Figure S3.

Point 6:

"Fig. 3D, both panels indicate Dephosphorylated proteins but maybe one of them should indicate KEGG with Phosphorylated proteins?"

Both KEGG analyses are now shown in Figure S4 .

Point 7:

" Fig. 7D, the restored accumulation of Erp1 upon combined depletion of Cdc27 and cyclin B3 is not very clear in the blot presented."

Experiment in Figure 7D has been repeated and the new western blot images shown.

Point 8:

"The model of Fig. S2 could be elaborated a bit more and included as a figure in the main manuscript".

The model in Figure S2 has been modified by adding a representation of the levels of cyclin B and the activity of APC in control and Gwl depleted oocytes. Due to the additional new data incorporated into the main figures in this revised version, we have retained the model as a supplementary figure. It can be moved to the main figures if the referee deems it essential.

Point 9:

"The choice of references is a bit odd and should be revised. For example, in page 6: "During meiotic progression, c-Mos is translated at GVBD promoting the activation of the MAP Kinase Kinase 1, MEK1, and the final phosphorylation of MAPK1 (ref. 17)". In fact, ref. 17 has

nothing to do with c-Mos translation at GVBD nor with MAPK1 activation in oocytes”.

We thank the referee for pointing out this error. Reference 17 has been modified.

Hoping for a positive answer

Best regards

Thierry Lorca
